# Design and synthesis of a chemically diverse, lead-like DNA-encoded library from sequential amide coupling

**DOI:** 10.1039/d5md00350d

**Published:** 2025-07-29

**Authors:** Cameron E. Taylor, Grace Roper, Rhianna Young, Fredrik Svensson, Andreas Brunschweiger, Sam Butterworth, Andrew G. Leach, Michael J. Waring

**Affiliations:** a Cancer Research Horizons Newcastle Drug Discovery Unit, Chemistry, School of Natural and Environmental Sciences, Newcastle University Bedson Building Newcastle upon Tyne NE1 7RU UK mike.waring@ncl.ac.uk; b Medical School, Newcastle University Framlington Place Newcastle upon Tyne NE2 4HH UK; c Cancer Research Horizons Therapeutic Innovation, Jonas Webb Building, Babraham Research Campus Cambridge CB22 3AT UK; d Institut für Pharmazie und Lebensmittelchemie, Lehrstuhl fur Pharmazeutische Chemie Am Hubland D-97074 Würzburg Germany; e School of Health Sciences, University of Manchester Oxford Road Manchester M13 9PL UK

## Abstract

DNA-encoded libraries (DELs) are established as an effective screening strategy to identify protein ligands and offer a cost-effective means of screening large numbers of compounds. However, the synthesis and utilisation of DELs is implemented by relatively few laboratories. Here, we describe the design and synthesis of a medium-sized DEL through simple amide coupling procedures. We provide details of chemistry and enzymatic steps and demonstrate their effectiveness by synthesising 300 thousand and 3 million-member DELs. We demonstrate their integrity through screening against carbonic anhydrase IX and show their chemical diversity through *in silico* comparison with an established high-throughput screening library. The DELs described can be used as a resource to accelerate hit identification for early-phase drug discovery and are available to the academic community for screening.

## Abstract 2

Accessible synthesis of DNA-encoded libraries of up to 3 million-members *via* sequential amide coupling, validated by CAIX screening. *In silico* analysis showed lead-like properties, and the libraries are available to support academic drug discovery.

## Graphical abstract

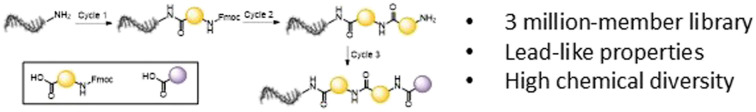

## Introduction

DNA-encoded libraries (DELs) have become a powerful hit-finding method in drug discovery,^1–4^ complementary to existing and emerging hit-identification techniques. Libraries potentially consisting of billions of encoded compounds can be synthesised, stored, and screened at significantly lower costs than HTS libraries due to the advantages of DNA encoding. This allows both combinatorial synthesis and the use of libraries as pooled mixtures. Signal amplification by PCR means that screening can be carried out on a very small scale without significant protein demands. Once robust chemistry is established, a large DEL can be synthesised in weeks and screened in days. As a result, DEL approaches are now employed by most large pharmaceutical companies as a core hit-finding capability and are accessible to others *via* specialist providers.^5–8^

Specialist equipment and techniques are required for DEL synthesis, and uptake in smaller companies and academic laboratories is limited partly for this reason. Preparation of very large libraries requires significant capabilities in reagent handling, information capture and logistics, as well as the cost associated with purchasing large numbers of specialised chemical building blocks and coding oligonucleotides. These high initial costs can be a barrier to those new to the field.

Although extremely large (>100 million-member) DELs are increasingly common, the advantages of larger libraries are perhaps overstated. Due to the lack of structural similarities in many commonly used reagent sets, the increase in diversity as the number of monomers is increased is limited. Furthermore, very large libraries have often relied on common scaffolds such as triazines,^9,10^ which further limits their structural diversity.^11–13^ Another drawback of large libraries is that it is harder to ensure library fidelity as size increases, as compromises have to be made in the validation of the chemical building block couplings. Selections from larger libraries can also be more challenging to sequence reliably since larger numbers of compounds increase signal noise and thus require significantly increased sequencing depth.^14^

Smaller, structurally simpler libraries can avoid some of these difficulties and are potentially more accessible. Chemical diversity can be sufficiently maintained with a judicious choice of DEL synthesis schemes and their constituent monomers. In particular, higher diversity results inherently from using library schemes that couple monomers together with minimal common substructures (rather than sequential additions to a central scaffold).

To explore these concepts further, we describe the development of a DEL from validation of building block couplings to library synthesis and selection against an exemplar protein, carbonic anhydrase IX (CAIX). We sought to design a lead-like library and investigate its quality by assessment of its physicochemical properties and chemical diversity in comparison with a high-quality traditional high-throughput screening library.

## Results and discussion

### Library design and validation

**Scheme 1:**
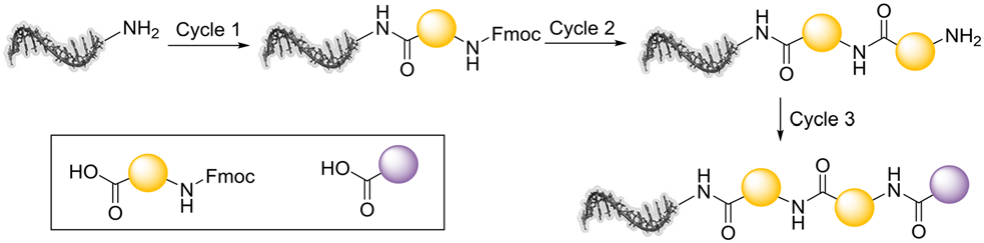
DEL synthesis scheme comprising two rounds of amide-coupled *N*-Fmoc amino acids with a final capping carboxylic acid.

A simple, linear, 3-cycle library design was chosen, utilising two readily available building block classes with well-established chemistry to rapidly synthesise a medium-sized DEL. This comprised two cycles of amide coupling of *N*-Fmoc-protected amino acids, each followed by Fmoc deprotection, followed by an amide coupling using capping carboxylic acids (Scheme 1). The amide coupling and Fmoc deprotection steps were optimised to work on a small scale with minimal use of reagents.

After a trial of amide coupling methods, we chose DMTMM-mediated reaction conditions, which gave good conversion across a range of monomers. Validation reactions using optimised conditions were performed in PCR plates using 250 pmol DNA with 630 equivalents of the acid (typically <100 μg per reaction (150 nmol)). This is significantly lower than most reported validation experiments, which use approximately 1.5 mmol.^15–18^ Completed reactions were diluted with water, and the crude mixture was analysed by RP-LCMS.

**Fig. 1:**
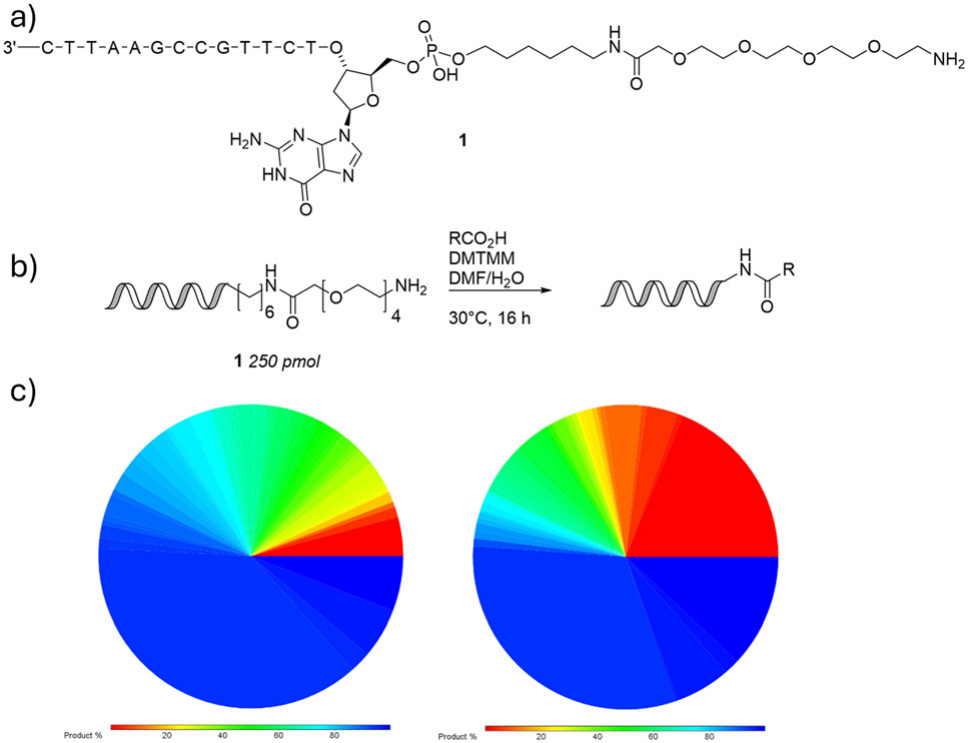
Summary of building block validations: a) 14 nt DNA headpiece 1 used as a model in this work; b) amide coupling validation reaction; c) observed conversions for *N*-Fmoc amino acids (left) and carboxylic acids (right) determined by AUC integration of RP-LCMS BPC (1050–3000 Da) chromatogram.

59 *N*-Fmoc amino acids and 116 carboxylic acids were selected based on chemical diversity, desirable functionality and physicochemistry (clog *P* and molecular weight). Each was evaluated for reaction efficiency by coupling to 14 nucleotide DNA headpiece 1 (Fig. 1). The validation experiments showed that a wide range of functional groups and acids were tolerated. Most unprotected aliphatic amines were incompatible as expected, however the majority of anilines coupled well. Alcohols and phenols proved problematic. The presence of very bulky groups α- to the carboxylic acid generally led to poorer conversions. It is hypothesised that the solubility of both the free carboxylate and activated ester play a significant role in determining conversion. However, some acids that were visibly sparingly soluble in DMF did couple with >95% conversion.

The deprotection of DNA-conjugated *N*-Fmoc protecting groups (using 10% aqueous piperidine) is well-documented and typically results in complete deprotection for a wide range of substrates.^15,19,20^ Our observations were consistent with the previous work. However, direct precipitation of the reaction mixture typically resulted in low recovery. Little is reported about this in the literature. To investigate isolation recovery, a model system using DNA-coupled-*N*-Fmoc alanine was used. It was supposed that the presence of piperidine was the major reason for the low recovery, so removal of the piperidine was attempted using centrifugal evaporation and buffer exchange *via* size exclusion centrifugal spin filtration.^21,22^ A combination of both methods was required for consistently high recovery (Table 1).

**Table 1:** Percentage recovery from 4 different purification methods after Fmoc deprotection (*n* = 3)

Conditions	Recovery%
Precipitation only	20 (±11)
Concentration then precipitation	49 (±5)
Buffer exchange, then precipitation	46 (±1)
Concentration, buffer exchange, then precipitation	89 (±2)

### Library synthesis

The validation data was used to guide building block selection, where high conversion was an important consideration for inclusion. We selected 55 *N*-Fmoc amino acids and 96 carboxylic acids for the library synthesis. Two control wells were included in each amide coupling step to simplify potential hit follow-up. The first was subjected to identical reaction conditions without the addition of any carboxylic acid, and in the second, no reagents were added. Including these controls identifies cases in which truncated side-products may contribute to activity observed in selections. DNA codons were designed with a Hamming distance of 3, and palindromic or hairpin-forming sequences were removed. 200 barcodes were designed for each cycle number. The full list of barcodes is detailed in the SI.

**Scheme 2:**
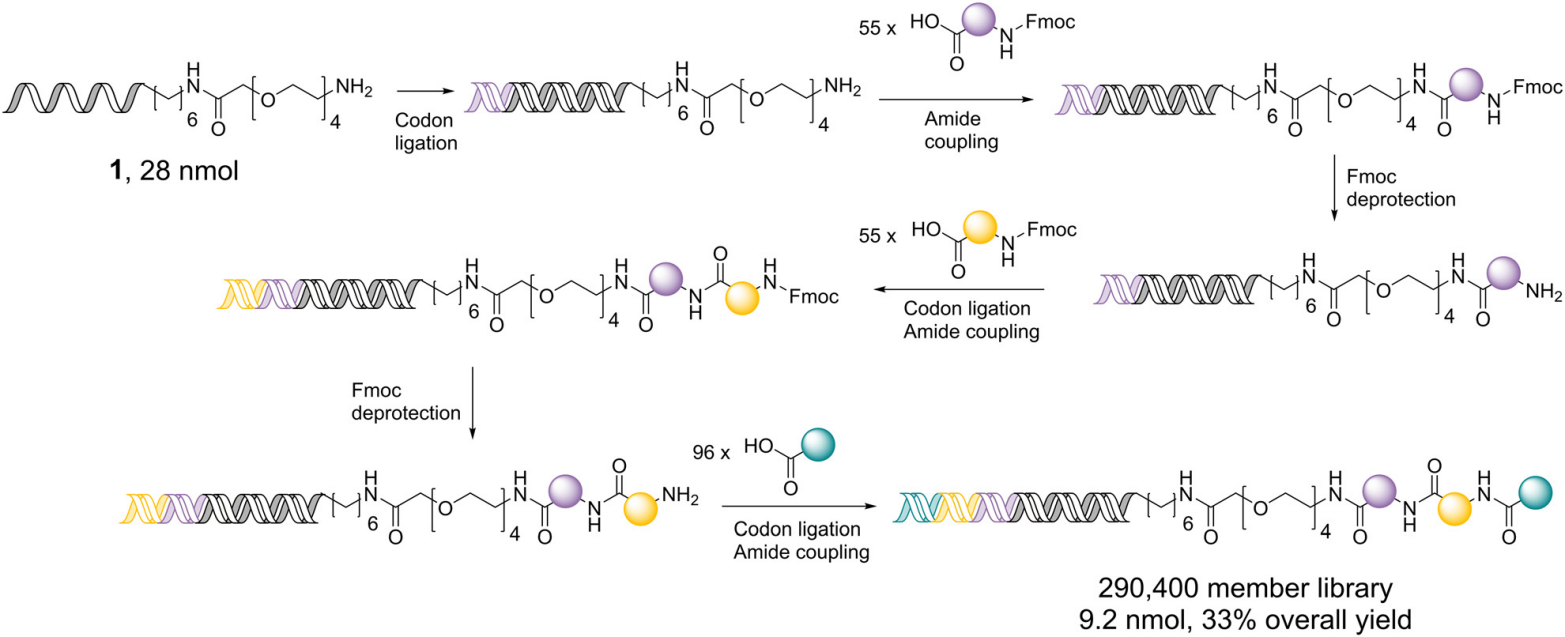
300 thousand DEL synthesis comprising two sequential cycles of coupling of 55 *N*-Fmoc amino acids (with Fmoc deprotection) followed by capping with 96 carboxylic acids.

Library synthesis began with the single-stranded DNA headpiece 1, which was subjected to two coupling cycles of the 55 *N*-Fmoc amino acids, each with subsequent Fmoc removal, followed by coupling of the 96 carboxylic acids (Scheme 2). An encoding step (ligation of the respective DNA codon sequences) was carried out prior to each amide coupling. The optimised chemistry conditions were used at the same concentration during library synthesis, which we believe to be an important measure to ensure library fidelity. Ligation efficiencies at each stage were assessed by analytical gel electrophoresis of a representative sample of wells (Fig. S7–S9). Precipitation of each ligation reaction was carried out in the plate. Avoiding transferring the samples to individual tubes helped maximise DNA recovery and reduced the risk of handling errors.^23^ The precipitate was pelleted by centrifugation, and the supernatant was removed prior to amide coupling in the same plate.

Overall, the final library was produced in 33% yield over the five synthesis and three encoding steps, resulting in 9.2 nmol of the final DEL. This library synthesis used far lower DNA input than the μmol quantities often used, making it an attractive starting point for new projects.^15–17,24^

### Selection against CAIX

**Fig. 2:**
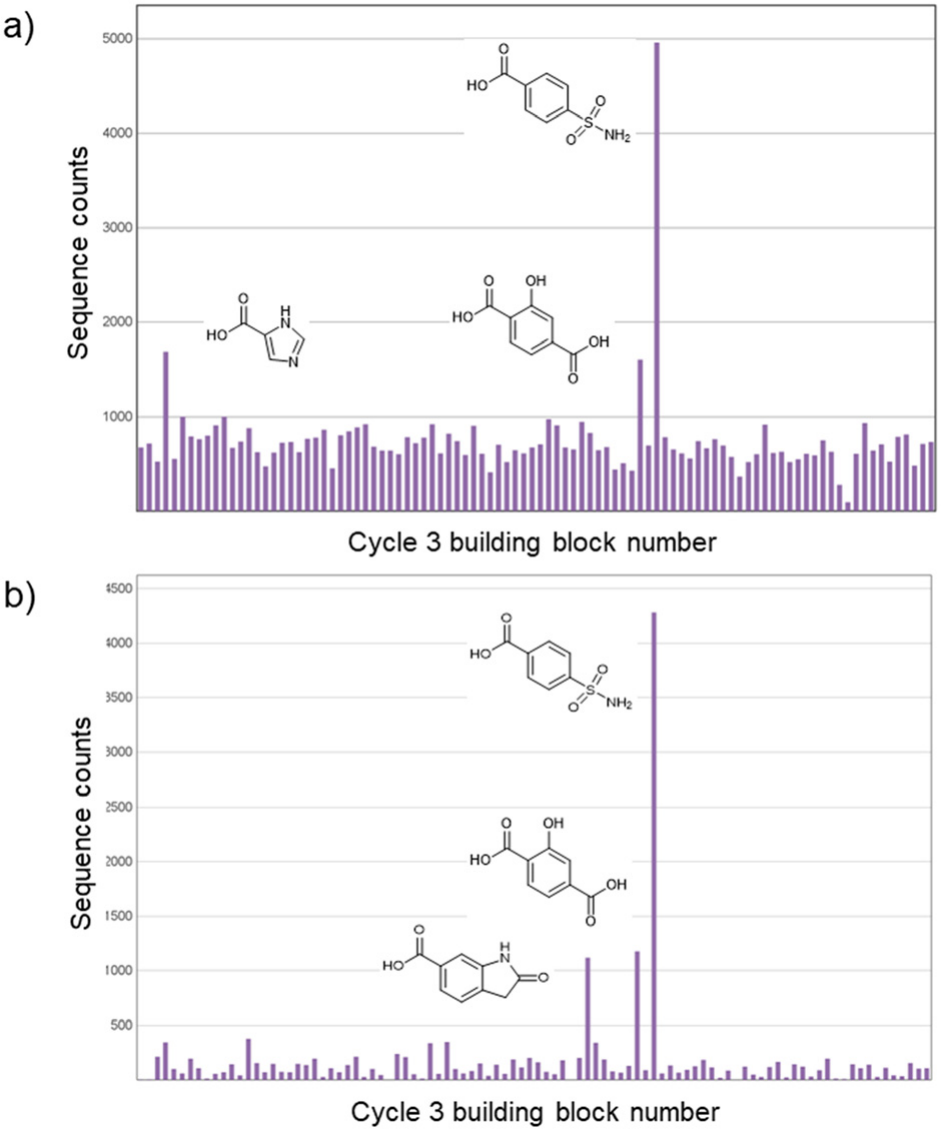
a) Sequence counts for the cycle 3 monomer for selections of the 300 thousand-member library against CAIX, a) initial selection, b) selections after pre-clearing the library against the beads.

The library was screened against CAIX as a test case. During affinity selections, we used approx. 1 million copies per compound (500 fmol library) and 4.8 μg His-tagged CAIX as reported by Kunig *et al.*^25–32^ Two rounds of selection were performed, and after PCR amplification and Illumina® sequencing, counts for unique DNA barcodes were summed for analysis. Analysis of the sequencing data revealed enrichment of 4-sulfamoylbenzoic acid from the cycle 3 building block set (Fig. 2a). To a lesser degree, imidazole-4-carboxylic acid and 2-hydroxybenzene-1,4-dicarboxylic acid were also enriched (Fig. 2a). There was no significant enrichment of the cycle 1 or 2 building blocks. Primary sulfonamides are well-precedented binding motifs in CAIX inhibitors, in which the sulfonamide group is known to interact directly with the catalytic zinc atom with high tolerance of other functionality within the ligand.^30^ Including this inbuilt control to the library confirms that the chemistry steps were successful and the building blocks were correctly encoded.

Imidazole-4-carboxylic acid was also enriched in selections against other targets using His-beads. Therefore, it was assumed that these compounds are false positives that are selected due to the imidazole binding to the NTA-cobalt-based beads directly. The addition of imidazole to block vacant bead sites and herring sperm DNA to outcompete non-specific DNA binding were both investigated. Adding imidazole did not eliminate the observed selection enrichment of the imidazole-containing compounds. However, the background noise was reduced when herring sperm DNA was used as a blocking agent.

As an alternative means to reduce the level of imidazole enrichment, the DEL was *pre-cleared* by incubating with a half portion of beads and the resulting supernatant was incubated with the immobilised protein. The pre-cleared library was selected against CAIX in the presence of herring sperm DNA, resulting in much lower imidazole enrichment and background noise, leading to a clearer trend for enrichment of the sulfonamide-containing compounds (Fig. 2b).

### Synthesis of a 3 million-member DEL

**Fig. 3:**
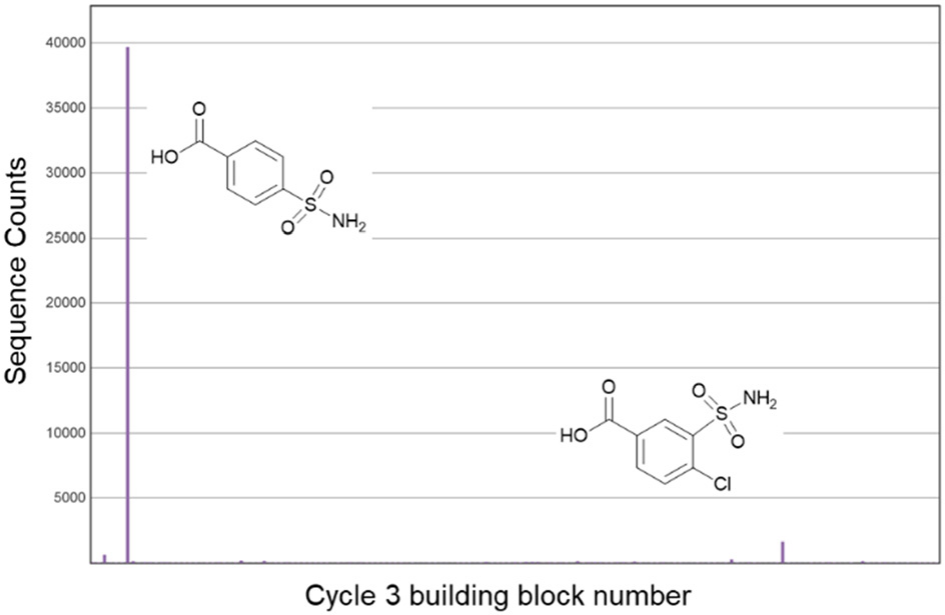
Sequence counts for the cycle 3 monomer for selection of the 3 million-member DEL against CAIX.

The same library scheme (Scheme 1) was expanded to produce a larger DEL. The building block validation experiments were expanded to include 171 *N*-Fmoc amino acids (after property filters). The validation of carboxylic acids was expanded to 227 carboxylic acids (SI). From this, 142 *N*-Fmoc amino acids and 147 carboxylic acids were included in the expanded library. The synthesis proceeded as expected and resulted in a yield of 9 nmol from an initial 56 nmol with good ligation efficiencies (Fig. S1–S3). The library was selected against His-CAIX with library pre-clearing and 4-sulfamoylbenzoic acid was clearly enriched (Fig. 3).

The 3 million-member DEL was assessed for its lead-like properties using established calculated descriptors and compared to the enamine HTS set, which was selected as a representative, high-quality screening library that is widely used in standard screening approaches. The DEL had overall very good lead-like properties with mean molecular weight (MWt) 438 Da, log *P* 1.7, hydrogen bond donors (HBD) 2.6, topographical surface areas (TPSA) 106, rotatable bond count (NRB) 7.4 and aromatic ring count (NAr) 1.8 (Table 2).^33–37^ These were similar to the enamine HTS library (the DEL is slightly higher in size overall and slightly more polar; no differences were significant).

**Table 2:** Mean and std. dev. values of calculated properties of the DEL (*n* = 3 089 664) and enamine HTS set (*n* = 1 367 257)

	DEL	HTS set
MWt	438 ± 69	334 ± 53
log *P*	1.7 ± 1.3	2.5 ± 1.1
HBD	2.6 ± 0.9	1.1 ± 0.8
TPSA	106 ± 19	69 ± 21
NRB	7.4 ± 2.2	4.5 ± 1.5
NAr	1.8 ± 1	2 ± 0.9

**Fig. 4:**
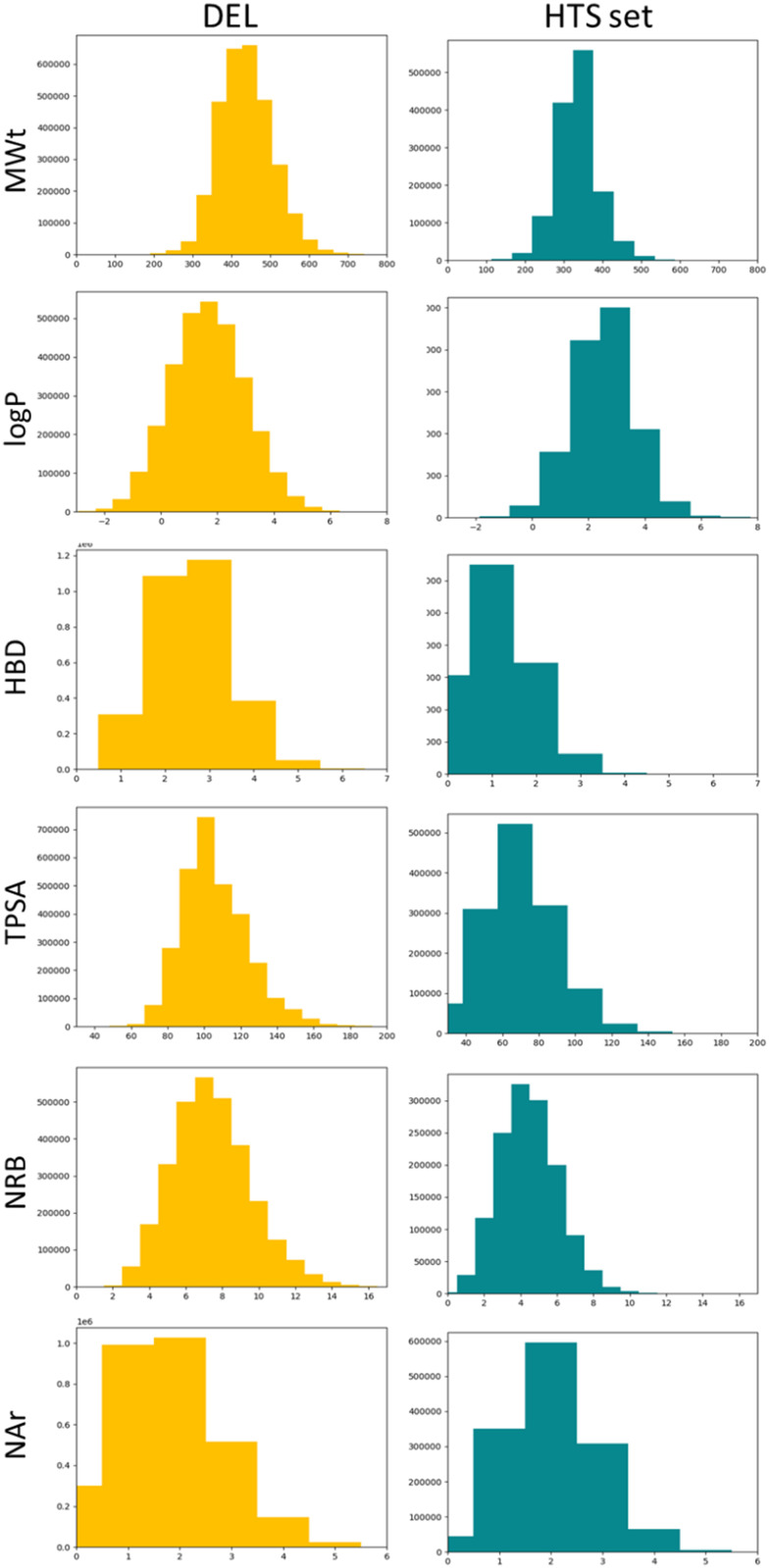
Calculated property distribution comparisons between the DEL (gold) and the enamine HTS set (teal).

Due to the combinatorial nature of DELs, it can be difficult to constrain physicochemical properties within a defined range. The combinations of the most extreme building blocks (the largest or most lipophilic in every cycle, for example) may lead to compounds in a DEL occupying a wider range of properties than traditional compound sets that consist of individually prepared sub-libraries, allowing tighter property distributions with fewer compounds outside the ideal range. In the case of this DEL, the property distributions were good overall. The DEL contained more MWt > 500 Da compounds than the HTS set, but this was not a significant portion of the total (Table 2 and Fig. 4). Log *P* and NAr distributions were similar between the two. HBD and PSA were slightly skewed to higher values in the DEL as a consequence of the inherent amide bonds arising from the synthesis, but the compounds generally populated the desirable range (Fig. 4).

**Fig. 5:**
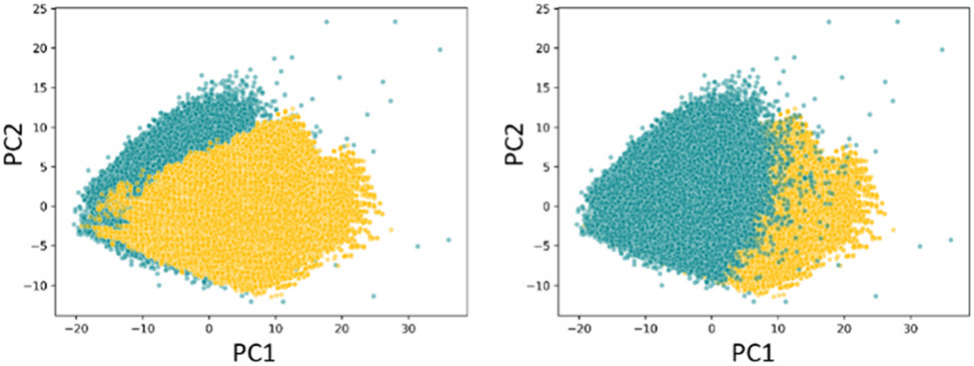
Chemical diversity principal components analysis of the DEL (gold) compared to the enamine HTS set (teal), left panel with DEL compounds on top, right panel with HTS compounds on top.

**Fig. 6:**
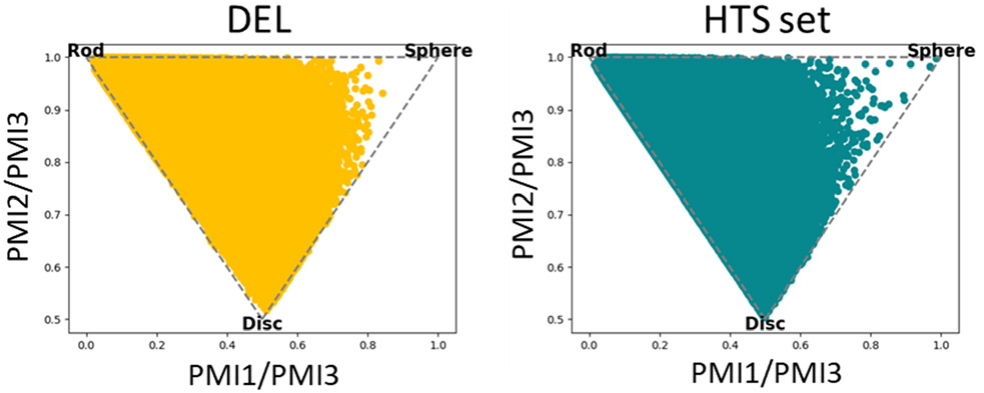
Principal moments of inertia plots for the DEL (gold) and the enamine HTS set (teal).

The chemical diversity of the DEL was assessed using a principal components analysis.^38^ The DEL had a similar diversity to the HTS with significant overlap in chemical space between the two sets and comparable areas populated exclusively with one or the other (Fig. 5). Principal moments of inertia (PMI) plots showed that the DEL compounds also have similar topology distributions to the HTS set with good representation across the majority of the plots (Fig. 6).^39^

## Conclusions

This work demonstrates the facile synthesis of a 3 million-member amide-coupled DNA-encoded library. The optimised library synthesis chemistry conditions are high-yielding and use μg quantities of monomers. These conditions, combined with the validated conversions for 171 *N*-Fmoc amino acids and 227 carboxylic acids, alongside the DNA barcodes, should be transferable to other libraries. This work will significantly reduce the time and cost required to initiate a similar DEL project. The selection of the synthesised libraries against CAIX demonstrates that the methodology for both the on-DNA chemistry and the corresponding encoding steps were successful. The selection conditions were optimised to reduce noise and the influence of bead-binding compounds; we believe the selection conditions presented will be useful as a general starting point for a DEL screening campaign against almost any immobilised target. Computational analysis showed that the DEL has good lead-like properties and chemical diversity comparable to a high-quality HTS set. This arises from using a library synthesis scheme that employs monomer couplings, not using a common scaffold, and due to the careful selection of monomers. This efficient and user-friendly library methodology will be of great utility to academic groups actively employing DEL technology and those looking to leverage DELs in their research. The DELs presented in this manuscript are available open-access for screening by academic users.

## Author contributions

CET carried out the DEL design and synthesis and the selections and co-wrote the manuscript, GR developed conditions for the selections, RY wrote the code that processes the library data, FS carried out the computational analysis of physicochemical properties and chemical diversity, AB cosupervised the project and designed the DNA sequences, SB cosupervised the project, AGL designed the DNA sequences, MJW supervised CET and GR, led the project and co-wrote the manuscript. All authors reviewed the manuscript prior to submission.

## Conflicts of interest

There are no conflicts to declare.

## Supplementary Material

MD-016-D5MD00350D-s001

MD-016-D5MD00350D-s002

MD-016-D5MD00350D-s003

MD-016-D5MD00350D-s004

## Data Availability

SI includes full experimental procedures, DNA-encoded library synthesis information, analytical data, NGS analysis information, and assay results. See DOI: https://doi.org/10.1039/D5MD00350D. Full experimental details and data supporting this article are available in the SI.
