# Supplementary material for: Design and synthesis of a chemically diverse, lead-like DNA-encoded library from sequential amide coupling
Source: RSC Med Chem. 2025 Jul 29;16(10):4774–80. doi: 10.1039/d5md00350d (PMC12333441; doi:10.1039/d5md00350d)
Supplement: MD-016-D5MD00350D-s001 [file MD-016-D5MD00350D-s001.pdf]

Supplementary Information (SI) for RSC Medicinal Chemistry.
This journal is © The Royal Society of Chemistry 2025

Supplementary Information

Design and synthesis of a chemically diverse, lead-like DNA-encoded library from sequential amide coupling

Cameron E. Taylor,[a] Grace Roper,[a] Rhianna Young,[b] Fredrik Svensson,[c] Andreas Brunschweiger,[d] Sam Butterworth,[e] Andrew G. Leach[e] and Michael J. Waring *[a]

Contents

Analytical and purification 2
Ion-pair chromatographic analysis of DNA conjugates by UPLC–MS 2
Preparative HPLC of oligonucleotide conjugates 2
Solvents, Reagents, and Proteins 2
Other methods and equipment 2
Headpiece synthesis 3
General methods: 3
Ethanol precipitation in tubes: 3
Ethanol precipitation after ligation in plates: 3
Phosphorylation 3
Building block validation acylation conditions 4
3M Library synthesis acylation conditions 4
Fmoc deprotection 4
Library Synthesis Overview: 4
3M-Member Library Ligations 5
Tandem ligation of forward primer binding site and BB1_code 5
BB2_code ligations 6
BB3_code ligations 7
PCR and NGS 8
Affinity selections 8
NGS Data Formatting 9
NGS Data Interpretation 10
Carbonic anhydrase assay 11
300k-Member library ligation gel electrophoresis images 12
Dynabeads™ M-280 Streptavidin 13
Selection of Acylation Conditions: 13

Analytical Data 15
References 26

Analytical and purification

Ion-pair chromatographic analysis of DNA conjugates by UPLC–MS

Conversions were estimated based on total ion count from negative ion mass spectrometry.[1]

Samples were resolved on an Agilent 1260 Infinity 2 system equipped with a C18 column (Agilent AdvanceBio Oligonucleotide, 2.1 x 100 mm, 2.7 µm, Part Number:655750-702) kept at 65 °C. DNA sample (1 - 5µL) was injected and eluted (40 –95% mobile phase B over 7.5 min, 0.45 mL/min flow rate; Mobile phase A: 25 mM HFIP / 15 mM HA in Milli-Q Water; Mobile phase B: MeOH.

DNA mass spectrometry was conducted on an Agilent 6550 iFunnel QTOF in negative mode, using a standard 3200 m/z maximum and a 2 GHz extended dynamic range; drying gas temperature was set to 260 °C at 12 L/min, sheath gas temperature was set to 350 °C at 12 L/min, nebuliser at 35 psig, VCap voltage of 4000 V and nozzle voltage of 2000 V.

Preparative HPLC of oligonucleotide conjugates

Samples were resolved on an Agilent 1260 Infinity system equipped with a C18 column (Phenomenex Clarity® Oligo-RP, 250 x 21.2 mm, 5.0 µm) at rt. DNA sample (50 - 250µL) was injected and eluted (2 –98% mobile phase B over 30 min, 20.0 mL/min flow rate; Mobile phase A: 50 mM HFIP / 15 mM DIPEA in Milli-Q Water; Mobile phase B: MeOH with UV monitoring at 254 nm.

Solvents, Reagents, and Proteins

Chemicals were purchased from Fluorochem, Sigma-Aldrich, Acros, Enamine, Liverpool ChiroChem, and Key Organics and were used without further purification. All water used with DNA substrates was nuclease-free (not DEPC-Treated). Solid supported 14-mer DNA strand was custom synthesised by Sigma-Aldrich. All other DNA building blocks were purchased in plates or tubes from IDT and provided as 100 µM solutions in pH 8.0 TE buffer or as dry pellets. C-His-tagged Human Carbonic Anhydrase IX / CA9 (38-414) was purchased from ACROBiosystems (CA9-H5226-50ug).

Other methods and equipment

Aqueous solutions of DNA were quantified spectrophotometrically with a Thermo Scientific Nanodrop One. Electrophoresis was performed with an Invitrogen E-GEL Power Snap device using commercial gels (E-Gel EX, 4% agarose) according to manufacturer guidelines, using Invitrogen™ E-Gel™ Ultra Low Range DNA Ladder (Product Code.15746548). Centrifugation was performed with an Eppendorf 5424R or Fisherbrand GT 2R. Centrifuge tubes used were Eppendorf snap cap, DNA LoBind®, 1.5 mL, Catalog No. 0030108051.

Headpiece synthesis

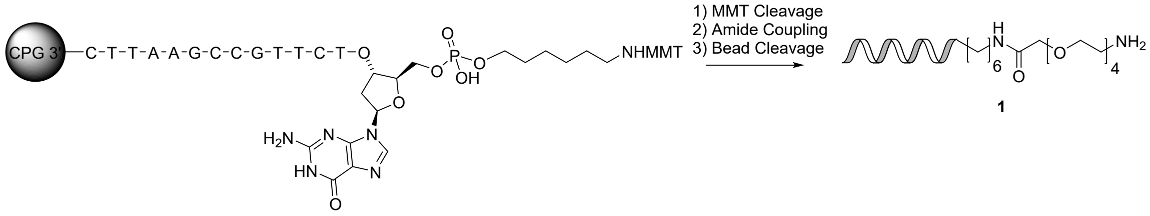

*Supplementary Scheme 1. Headpiece **1** synthesis*

Step 1: The MMT-protecting group of the CPG-bound oligonucleotide (50 mg of solid phase material) was removed by washing with a total of 10 mL 3% (0.6 g/20 mL) trichloroacetic acid in CH_2Cl_2 over approx. 2 h. An orange colouring of the solution indicated successful removal of the protecting group. The CPG-bound deprotected DNA was then washed with 10 mL DCM over approx. 1 h, and left to air dry.

Step 2: To a 1.5 mL tube was added 19 mg 1-(9Hfluoren-9-yl)-3-oxo-2,7,10,13,16-pentaoxa-4-azaoctadecan-18-oic acid. To this was added 17 mg HATU, 17 µL Hunig's base, 1.0 mL DMF, and the mixture was shook at 600 rpm for 20 min rt. To this mixture, add the CPG-bound deprotected DNA and shake (600 rpm) rt overnight. The CPG solid phase was then filtered over a filter column, and washed subsequently with 3 x 500 µL DMF, MeCN, MeOH, and CH_2Cl_2, then left to air dry.

Step 3: The bead cleavage of the oligonucleotide conjugate was performed via the addition of 500 µL 40% aq $MeNH_2$ and 500 µL 30% aq NH_4OH and the mixture was shook at 600 rpm for 2 h rt. The CPG solid was filtered over a filter column, and washed with IDTE buffer 3 × 500 µL. The crude was then purified by preparative HPLC.

General methods:

Ethanol precipitation in tubes:

To a given volume (100% v/v), aq. NaCl (5 M, 10% v/v) and EtOH (275% v/v) were added. The reaction vessel was briefly vortexed and incubated at −20 °C for 16 h. After centrifugation (25 min, 21130 rcf, 4 °C), the supernatant was aspirated. To wash the pellet, 70% aq. EtOH (175-200% v/v) was added. The vessel was then immediately centrifuged (10 min, 21130 rcf, 4 °C), and the supernatant was aspirated. The pellet was then left to air dry.

Ethanol precipitation after ligation in plates:

To a given volume (100% v/v), aq. NaCl (5 M, 10% v/v) and EtOH (275% v/v) were added. The plate was incubated at −20 °C for 16 h. After centrifugation (1 h, 2272 rcf, 4 °C), the supernatant was aspirated. The pellet was then left to air dry.

Phosphorylation

Prior to ligation, the 5' terminus of each DNA strand that required ligating was phosphorylated.

DNA (1 nmol) was dried in a 96-well plate (MicroAmp™ Optical 96-Well Reaction Plate Catalog number: N8010560) using a Genevac™ Concentrator EZ-2 Plus, and a freshly prepared phosphorylation mixture* (20 µL) was then added. The plate was sealed with heat-sealing foil, spun down, and incubated at 37.0 °C for 1 h, then heat denaturated at 75.0 °C for 12 min using an Applied Biosystems™ ProFlex™ PCR System, 96-well (cover temp 75.0 °C). Phosphorylated DNA was used in subsequent ligations without purification or precipitation. This protocol was directly scaled depending on the DNA amount.

*Phosphorylation mixture was composed of:

2 µL of 10X Reaction Buffer A (Thermo Scientific, Catalog number: EK0032), 2 µL of 10 mM ATP (diluted from 100 mM ATP Solution, Thermo Scientific, Catalog number: R0441), 15 µL nuclease-free water (Fisher Scientific, Product Code.10793837), and 1 µL of T4 Polynucleotide Kinase (10 U/µL) (Thermo Scientific, Catalog number: EK0032).

Building block validation acylation conditions

DNA (250 pmol) was dried in a 96-well plate (MicroAmp™ Optical 96-Well Reaction Plate Catalog number: N8010560) using a Genevac™ Concentrator EZ-2 Plus, and subsequently dissolved in 2.5 µL borate buffer (150 mM, pH 9.4). DMTMM (0.38 µL, 250 mM (aq), freshly prepared) and carboxylic acid solution (0.63 µL, 250 mM DMF) were added and the plate was sealed with heat-sealing foil, spun down, and incubated at 30 °C for 18 h using a Applied Biosystems™ ProFlex™ PCR System, 96-well (cover temp 105 °C). The plate was then spun down and a 2nd portion of fresh DMTMM solution was added. The plate was sealed with heat-sealing foil, spun down, and incubated at 30 °C for 4 h. The plate was then spun down and 6.5 µL H_2O was added prior to mass spectrometry analysis.

3M Library synthesis acylation conditions

DNA (250 pmol) was dried in a 96-well plate (MicroAmp™ Optical 96-Well Reaction Plate Catalog number: N8010560) using a Genevac™ Concentrator EZ-2 Plus, and subsequently dissolved in 2.5 µL borate buffer (150 mM, pH 9.4). DMTMM (0.38 µL, 250 mM (aq), freshly prepared) and carboxylic acid solution (0.63 µL, 250 mM DMF) were added and the plate was sealed with heat-sealing foil, spun down, and incubated at 30 °C for 18 h using a Applied Biosystems™ ProFlex™ PCR System, 96-well (cover temp 105 °C). The plate was then spun down and a 2nd portion of fresh DMTMM solution was added. The plate was sealed with heat-sealing foil, spun down, and incubated at 30 °C for 4 h. The plate was then spun down and each well was rinsed twice with 60 µL TE buffer. The combined washes were concentrated via centrifugal filtration using Millipore Amicon Ultra 0.5 mL – 3kDa cutoff (according to manufacturer guidelines) prior to ethanol precipitation.

Fmoc deprotection

DNA was reconstituted to 50 µM in water in a centrifuge tube. Piperidine was added to a final solution of 10% v/v piperidine. The sample was then placed in a Starlab Thermomixer-Mixer HC Art. No. S8012-0000 at 600 rpm, 37 °C, 1 h. The sample was then dried in a Genevac™ Concentrator EZ-2 Plus at 43 °C for 30 min before adding 200 µL TE buffer and centrifugal filtration using Millipore Amicon Ultra 0.5 mL – 3kDa cutoff (according to manufacturer guidelines) prior to ethanol precipitation.

Library Synthesis Overview

Library synthesis used split and pool methodology in 96-well plates, encoding each step prior to building block coupling. Briefly, DNA oligos were phosphorylated prior to tandem ligation of the forward primer binding site and BB1_code to the DNA headpiece, followed by ethanol precipitation in the plate. Acylation with each individual *N*-Fmoc amino acid building block was performed in the 96-well plate. The library was then pooled, precipitated using ethanol, followed by *N*-Fmoc deprotection and a final ethanol precipitation to finish the first cycle of synthesis. This process was repeated to incorporate a 2nd set of *N*-Fmoc amino acid building blocks, and a 3rd time to incorporate a set of carboxylic acid building blocks (without *N*-Fmoc deprotection in this cycle).

3M-Member Library Ligations

Tandem ligation of forward primer binding site and BB1_code

Target structures:

BB1 code OH1 Library codon FWD Primer 14mer

3’ NNNNNNNNNN TATG AATTGTATCA GTTTAGGCAAGTGTGGCTGGA CTTAAGCCGTTCTG 5’

5’ TAGG NNNNNNNNNN ATAC TTAACATAGT CAAATCCGTTCACACCGACCT GAATTCGGCAAGAC 3’

DNA codes (390 pmol; 1.00 eq) were added to each BB1 code (390 pmol; 1.00 eq) phosphorylation mixture in a MicroAmp™ Optical 96-Well Reaction Plate (Catalog number: N8010560) as follows:

The forward primer binding site and its complementary sequence were added as crude phosphorylation mixtures (1.56 µL). The DNA HP was added as a solution in TE buffer (1.02 µL, 0.38 mM). The appropriate BB1 complementary code was added as a solution in TE buffer (3.90 µL, 100 µM).

The mixture was then annealed by heating to 85.0 °C for 10 min, then slowly cooled to 4.0 °C using a Applied Biosystems™ ProFlex™ PCR System, 96-well (cover temp 105 °C).

A freshly prepared ligation mixture* (19.26 µL) was then added. The plate was sealed with heat-sealing foil, spun down, and incubated at 25.0 °C for 16 h, then heat denaturated at 75.0 °C for 12 min using a Applied Biosystems™ ProFlex™ PCR System, 96-well (cover temp 105 °C).

*Ligation mixture was composed of:

3.51 µL of 10× ligation buffer (Thermo Scientific, T4 DNA Ligase Buffer (10X), Catalog number: B69), 15.16 µL nuclease-free water (Fisher Scientific, Product Code.10793837), and 0.59 µL of T4 DNA Ligase, HC (30 U/µL) (Thermo Scientific, Catalog number: EL0013).

A 0.2 µL sample was taken from 7 representative wells, diluted in 19.80 µL of water and analysed using an Invitrogen E-GEL Power Snap as described above.

Ligations were observed to be complete; with a solid band at approx. 59 base pairs relative to the ladder. Results are shown in Supplementary Fig.1. All crude ligations (after sampling) underwent EtOH plate precipitation.

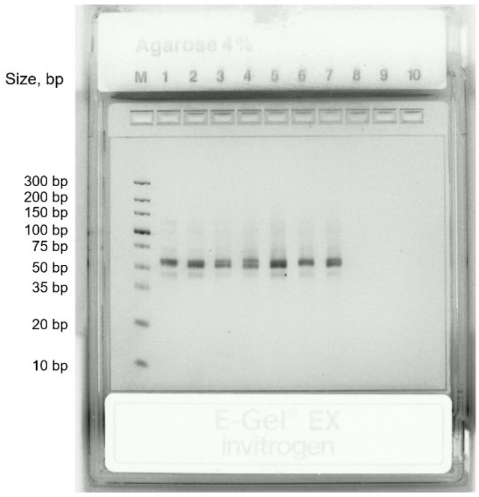

Supplementary Fig. 1 Analysis of tandem forward primer binding site and BB1_code ligation products by 4% agarose electrophoresis. The gel images represent single experiments; the experiments were not repeated.

BB2_code ligations

Target structures:

BB2 code OH2 BB1 code OH1 Library codon FWD Primer 14mer

3' NNNNNNNNNN ATCC NNNNNNNNNN TATG AATTGTATCA GTTTAGGCAAGTGTGGCTGGA CTTAAGCCGTTCTG 5'

5' CGTA NNNNNNNNNN TAGG NNNNNNNNNN ATAC TTAACATAGT CAAATCCGTTCACACCGACCT GAATTCGGCAAGAC 3'

DNA codes (105 pmol; 1.00 eq) were added to each BB2 code (105 pmol; 1.00 eq) phosphorylation mixture in a MicroAmp™ Optical 96-Well Reaction Plate (Catalog number: N8010560) as follows:

The cycle 1 library was added as crude phosphorylation mixture (1.51 µL). The appropriate BB2 complementary code was added as a solution in TE buffer (1.05 µL, 100 µM).

The mixture was then annealed by heating to 85.0 °C for 10 min, then slowly cooled to 4.0 °C using a Applied Biosystems™ ProFlex™ PCR System, 96-well (cover temp 105 °C).

A freshly prepared ligation mixture* (4.79 µL) was then added. The plate was sealed with heat-sealing foil, spun down, and incubated at 25.0 °C for 16 h, then heat denatured at 75.0 °C for 12 min using a Applied Biosystems™ ProFlex™ PCR System, 96-well (cover temp 105 °C).

*Ligation mixture was composed of:

0.95 µL of 10× ligation buffer (Thermo Scientific, T4 DNA Ligase Buffer (10X), Catalog number: B69), 3.68 µL nuclease-free water (Fisher Scientific, Product Code.10793837), and 0.16 µL of T4 DNA Ligase, HC (30 U/µL) (Thermo Scientific, Catalog number: EL0013).

A 0.2 µL sample was taken from 7 representative wells, diluted in 19.80 µL of water and analysed using an Invitrogen E-GEL Power Snap as described above.

Ligations were observed to be complete, with no detectable cycle 1 library (59 bp) remaining and a solid band at approx. 73 base pairs relative to the ladder. Results are shown in Supplementary Fig.2. All crude ligations (after sampling) underwent EtOH plate precipitation.

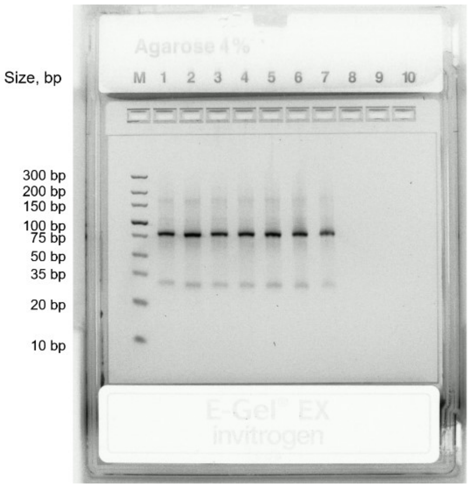

Supplementary Fig. 2 Analysis of BB2_code ligation products by 4% agarose electrophoresis. The gel images represent single experiments; the experiments were not repeated.

BB3_code ligations

Target structures:

REV Primer BB3 code OH3 BB2 code OH2 BB1 code OH1 Library codon FWD Primer 14mer

3’ ACATCTGGTACATCAACTCCAGT NNNNNNNNNN GCAT NNNNNNNNNN ATCC NNNNNNNNNN TATG AATTGTATCA GTTTAGGCAAGTGTGGCTGGA CTTAAGCCGTTCTG 5’

5’ TGTAGACCATGTAGTTGAGGTCA NNNNNNNNNN CGTA NNNNNNNNNN TAGG NNNNNNNNNN ATAC TTAACATAGT CAAATCCGTTCACACCGACCT GAATTCGGCAAGAC 3’

DNA codes (68 pmol; 1.00 eq) were added to each BB3 code (68 pmol; 1.00 eq) phosphorylation mixture in a MicroAmp™ Optical 96-Well Reaction Plate (Catalog number: N8010560) as follows:

The cycle 2 library was added as crude phosphorylation mixture (0.98 µL). The appropriate BB3 complementary code was added as a solution in TE buffer (0.68 µL, 100 µM).

The mixture was then annealed by heating to 85.0 °C for 10 min, then slowly cooled to 4.0 °C using a Applied Biosystems™ ProFlex™ PCR System, 96-well (cover temp 105 °C).

A freshly prepared ligation mixture* (5.34 µL) was then added. The plate was sealed with heat-sealing foil, spun down, and incubated at 25.0 °C for 16 h, then heat denatured at 75.0 °C for 12 min using a Applied Biosystems™ ProFlex™ PCR System, 96-well (cover temp 105 °C).

*Ligation mixture was composed of:

0.90 µL of 10× ligation buffer (Thermo Scientific, T4 DNA Ligase Buffer (10X), Catalog number: B69), 4.29 µL nuclease-free water (Fisher Scientific, Product Code.10793837), and 0.15 µL of T4 DNA Ligase, HC (30 U/µL) (Thermo Scientific, Catalog number: EL0013).

A 0.2 µL sample was taken from 7 representative wells, diluted in 19.80 µL of water and analysed using an Invitrogen E-GEL Power Snap as described above.

Ligations were observed to be complete, with no detectable cycle 2 library (73 bp) remaining and a solid band at approx. 110 base pairs relative to the ladder. Results are shown in Supplementary Fig.3. All crude ligations (after sampling) underwent EtOH plate precipitation.

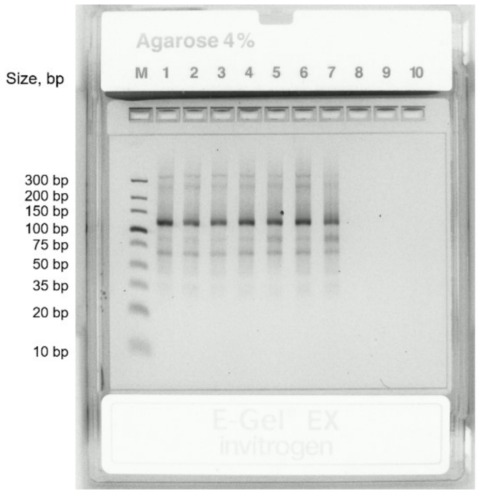

Supplementary Fig. 3 Analysis of BB3_code ligation products by 4% agarose electrophoresis. The gel images represent single experiments; the experiments were not repeated.

PCR and NGS

Barcodes of a DEL sample (input or eluted after screening) were PCR amplified using 1 µL forward extension primer (5'-ACACTCTTTCCCTACACGACGCTCTTCCGATCTTGTAGACCATGTAGTTGAGGTCA-3')(10 µM stock in TE buffer), 1 µL reverse extension primer (5'-GACTGGAGTTCAGACGTGTGCTCTTCCGATCTAGGTCGGTGTGAACGGATTTG-3')(10 µM stock in TE buffer), 20 µL DEL sample, 3.0 µL nuclease-free water (Fisher Scientific, Product Code.10793837), and 25 µL AmpliTaq Gold™ 360 Master Mix (Thermo Scientific, Catalog number: 4398886). The PCR method is as follows: 95 °C for 10 min; 38 cycles of 95 °C (30 s), 55 °C (30 s), 72 °C (60 s); 72 °C for 7 min; hold at 10 °C. The PCR products were cleaned up using NucleoSpin Gel and PCR Clean-up Columns for gel extraction and PCR clean up (Macherey-Nagel, Item number: 740609.250) (according to manufacturer guidelines). The DNA concentration was quantified spectrophotometrically, then sequencing reactions and adapter sequences trimming were conducted at Genewiz (Leipzig, Germany). The pooled DNA libraries were loaded on the Illumina instrument according to manufacturer's instructions. The samples were sequenced using a 2x 250 paired-end (PE) configuration. Image analysis and base calling were conducted by the Illumina Control Software on the Illumina instrument. The raw Illumina reads were checked for adapters and quality via FastQC. The raw Illumina sequence reads were trimmed of their adapters using Trimmomatic v. 0.36. Raw sequence data (.bcl files) generated from Illumina MiSeq were converted into fastq files and de-multiplexed using Illumina bsl2fastq v. 2.17 program.

Affinity selections

The protein targets and bead-only controls were screened via the same protocol. Dynabeads™ His-Tag Isolation and Pulldown (Invitrogen™ 10103D) or Dynabeads™ M-280 Streptavidin (Invitrogen™ 11205D) were transferred to a 1.5 mL centrifuge tube and placed on a magnetic rack. Beads were washes four times with 100 µl PBS buffer (+ 0.01% Tween® 20) then washed 2 times with 100 µl immobilisation buffer (protein buffer + 0.01% Tween® 20). Afterwards, 10 µl of protein (4.8 µg) was added and incubated with the beads for 30 min at 4 °C with continuous gentle mixing. The beads were then washed two times with 100 µL immobilisation buffer, one time with 100 µL bead blocking buffer and again two times with 100 µL immobilisation buffer. Afterwards, 50 µL of DEL was incubated with the protein-coated beads for 45 min at rt with continuous gentle mixing. The beads were then washed 8 times with 100 µL immobilisation buffer. DNA conjugates were eluted by heat

denaturation from the protein (80 °C, 5 min). Heat denaturation was performed two times in 20 µL ddH$_2$O. Subsequent rounds of the affinity assay were performed as described above using 20 µL of the eluted DNA conjugates and 30 µL of immobilisation buffer.

NGS Data Formatting

NGS files (.fastq.gz) were formatted for analysis using the following Linux script, which was written in-house.

gzip -d **INPUT_NAME.fastq.gz** && mv **INPUT_NAME.fastq INPUT_NAME.csv** && awk 'NR % 4 == 2' **INPUT_NAME.csv** |awk '{printf "%s,%s,%s,%s,%s,%s,%s,%s,%s\n", substr($0,1,21), substr($0,22,10), substr($0,32,4), substr($0,36,10), substr($0,46,4), substr($0,50,10), substr($0,60,4), substr($0,64,10), substr($0,74)}' |cut -f 2,4,6,8 --delimiter=',' | awk -F, '{print $0 "," $2 "; " $3 "; " $4}' | awk -F, '{if (c1[$1,$5]++ > 0) c[$1,$5]++; l[NR]=$0} END{for(i=0;i++<NR;){split(l[i],s,",");print l[i]","c[s[1],s[5]]}}' | awk -F, '{$6=$6+1;print}' OFS=, | awk -F',' '!a[$1,$5]++' > **OUTPUT_NAME.csv** && sed -i -e '1i"Library","BB1","BB2","BB3","Concat Barcode","Count"' **OUTPUT_NAME.csv**

<u>Script Breakdown</u>

Unzip the file.

Note: the original zipped file will be replaced – have a back up saved.

gzip -d **INPUT_NAME.fastq.gz**

Convert the file to a csv just by renaming the file.

mv **INPUT_NAME.fastq INPUT_NAME.csv**

Remove lines other than the 2nd line in every 4 lines (isolate sequence)

awk 'NR % 4 == 2' **INPUT_NAME.csv**

Break the lines up at different intervals. This will create 9 columns corresponding to: Primer, Library, OH1, BB1, OH2, BB2, OH3, BB3, Surplus (separated by commas).

Example: %s denotes number of substrings. ($0,50,10) means to extract a substring of length 10, starting from the 50th character of the input record.

awk '{printf "%s,%s,%s,%s,%s,%s,%s,%s,%s\n", substr($0,1,21), substr($0,22,10), substr($0,32,4), substr($0,36,10), substr($0,46,4), substr($0,50,10), substr($0,60,4), substr($0,64,10), substr($0,74)}'

Removes columns other than 2, 4, 6 and 8. (Library, BB1, BB2, BB3).

cut -f 2,4,6,8 --delimiter=','

Concatenates contents of columns 2, 3 and 4, separated by a semi colon, and adds this to a new column.

awk -F, '{print $0 "," $2 "; " $3 "; " $4}'

Counts duplicates. Duplicates must have the same contents (independently) in column 1 (library) and column 5 (concat barcode). Count is added on as another column.

Note: *count is for duplicates, not occurrences, so starts from 0 not 1.*

awk -F, '{if (c1[$1,$5]++ > 0) c[$1,$5]++; l[NR]=$0} END{for(i=0;i++<NR;){split(l[i],s,",");print l[i]",",c[s[1],s[5]]}}'

Adds 1 to every value in column 6. This should now reflect every count.

awk -F, '{$6=$6+1;print}' OFS=,

Removes duplicates that match column 1 and 5 independently. This is the last command in a pipe so save as new file name.

awk -F',' '!a[$1,$5]++' > **OUTPUT_NAME.csv**

Add column headings to the final file.

sed -i -e '1i"Library","BB1","BB2","BB3","Concat Barcode","Count"' **OUTPUT_NAME.csv**

Commands are put together using pipe |, which carries the input of one pipe over to the next, and && which carries out a command directly after another if the first is successful.

Once formatted, the NGS count data was then merged with a csv file containing SMILES structures of the library building blocks and the associated encoding DNA barcodes. Data which did not correspond to known library codes was discounted.

NGS Data Interpretation

To avoid potential bias and errors, the sequencing data presented in the main text refer to raw counts only. However, alternative methods of data interpretation were also interrogated, including enrichment of normalised percentage count over the no protein negative control, or enrichment of normalised percentage count over the blank library. All three approaches (raw counts, enrichment over no protein, and enrichment over blank library) showed clear enrichment of the sulfonamide-containing BB3 against CAIX.

Normalisation: Percentage Count = Count/(Total Number of Counts) × 100

Enrichment Sum = (Percentage Count R2) - (Percentage Count Beads)

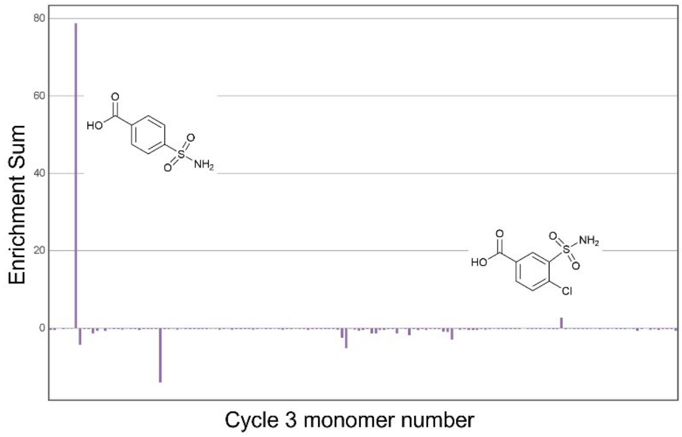

Supplementary Fig. 4 Normalised enrichment sum vs no protein for the cycle 3 monomer for selection of the 3M member DEL against CAIX.

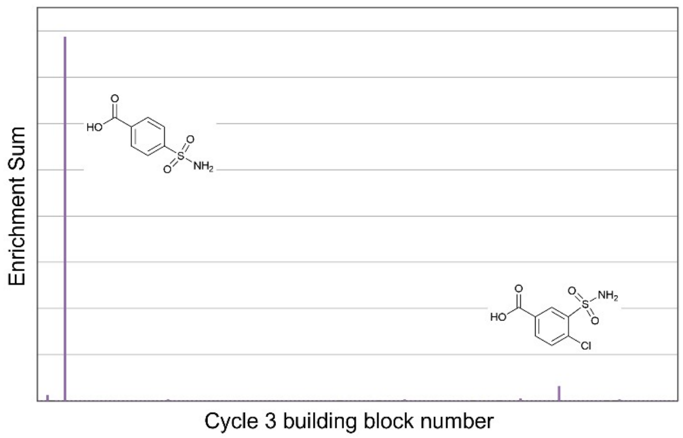

Supplementary Fig. 5 Normalised enrichment sum vs blank library for the cycle 3 monomer for selection of the 3M member DEL against CAIX.

Carbonic anhydrase assay

To validate that the selected hits were genuine, one of the enriched library members was selected for off-DNA synthesis. The most enriched compound, which resulted from the coupling of 4-sulfamoylbenzoic acid with S-N-methyl phenyl alanine (cycle 2) and S-2-cyclopropylglycine (cycle 1), was selected for synthesis as its methylamine capped derivative SI1. Testing of compound SI1 in a colourimetric assay for CAII revealed it to be a 425 nM inhibitor.[2]

Carbonic anhydrase II (CAII) dose-response inhibition assays were performed using a commercially available kit (BioVision, #K473-100), following the manufacturer's protocol, in a clear 96-well, F-bottom, low-binding, microplate (Greiner Bio-One, #655901). Compounds were tested at ten concentrations (half-log dilutions from 100 µM to 3 nM), with three technical replicates performed. A DMSO control, a known inhibitor control (acetazolamide, 200 µM), no-enzyme control for each top compound concentration, and a no enzyme with no compound control were each performed with three technical replicates. Absorbance at 405 nm was read on a BMG PHERAstar FSX every 30s, shaking for 5s before each reading.

A 10 minute pre-incubation of enzyme and inhibitors was performed, adding 10 µL of compound (prepared in 10% DMSO, 90% kit buffer), 80 µL of buffer, and 5 µL of enzyme (or buffer) to each well. The assay was initiated by addition of 5 µL substrate to each well, and the plate was immediately put in the PHERAstar and absorbance measurements commenced.

Percentage inhibition was calculated, by comparing the normalised slope of change in absorbance over time to the DMSO control, in the linear range of CAII activity. Percentage inhibition was plotted *versus* log(inhibitor concentration) in GraphPad Prism v10.1.2, and dose-response curves were fitted using nonlinear regression to give IC_{50} values.

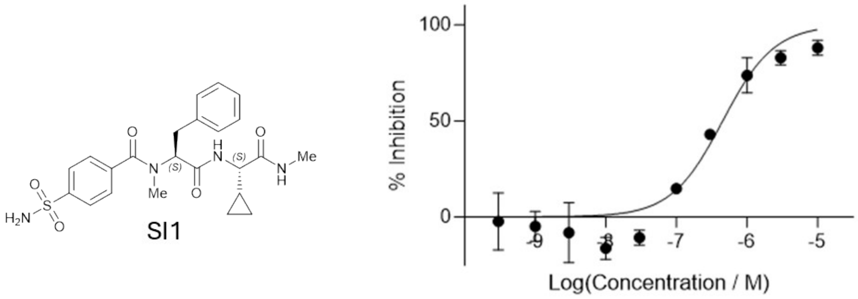

Supplementary Fig. 6 Off DNA-validation of CA activity of selected top hit SI1 in colourimetric CA dose-response assay results (N = 3 technical replicates, average ± S.D. shown).

300k-Member library ligation gel electrophoresis images

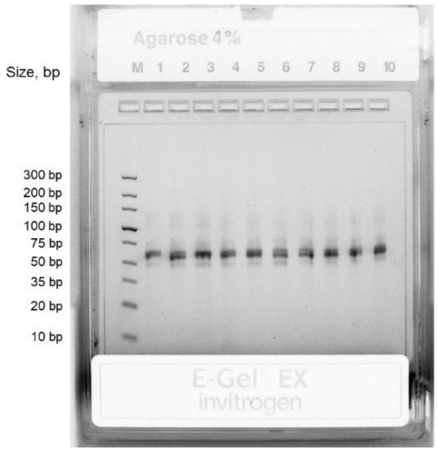

Supplementary Fig. 7 Analysis of tandem forward primer binding site and BB1_code ligation products by 4% agarose electrophoresis. The gel images represent single experiments; the experiments were not repeated.

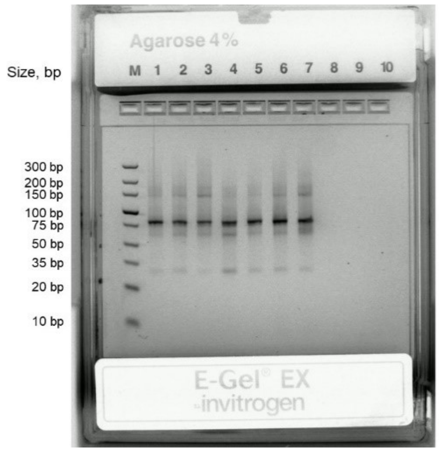

Supplementary Fig. 8 Analysis of BB2_code ligation products by 4% agarose electrophoresis. The gel images represent single experiments; the experiments were not repeated.

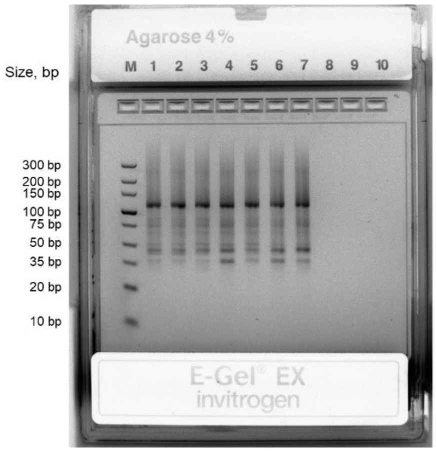

Supplementary Fig. 9 Analysis of BB3_code ligation products by 4% agarose electrophoresis. The gel images represent single experiments; the experiments were not repeated.

Dynabeads™ M-280 Streptavidin

Non-specific enrichment of specific BB3 molecules had been identified when using Dynabeads™ M-280 Streptavidin. In particular, enrichment of pyridone capping acid (and the linker *N*-Fmoc AA) was observed when using streptavidin beads (Figure SI10).

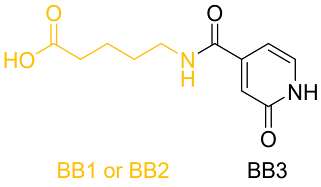

Supplementary Fig. 10 Structures of Dynabeads™ M-280 Streptavidin binders observed.

To reduce background noise and non-specific pyridone enrichment, we tested four different bead blocking buffers: PBST without supplements and PBST supplemented with either 100 µM biotin, 0.2 mg/mL herring sperm DNA, or 0.1 mg/mL BSA. The trend in pyridone enrichment decreased when biotin was used as a blocking agent. Additionally, significantly lower background noise was observed when herring sperm was used as a blocking agent for the beads and during DEL incubation. The combination of 100 µM biotin as a bead blocking agent and herring sperm DNA during DEL incubation significantly reduced the number of identified compounds compared to using biotin alone.

Selection of Acylation Conditions:

Our laboratory has extensive experience using a wide range of amide coupling conditions for on-DNA chemistry. In our hands, DMTMM-mediated coupling conditions are both operationally simple and provide good conversion across a broad range of building blocks. A representative comparison is shown below.

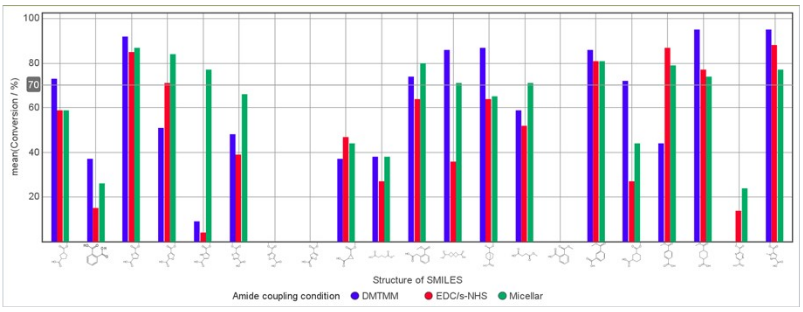

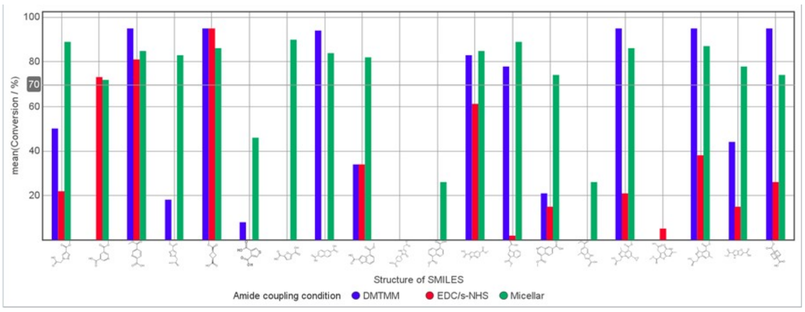

Supplementary Fig. 11 Exemplar comparison of amide-coupling conditions

Analytical Data

Synthesis of DNA Headpiece **1**: **MS** (ESI-) expected 4639.9 Da, found 4640.0 Da

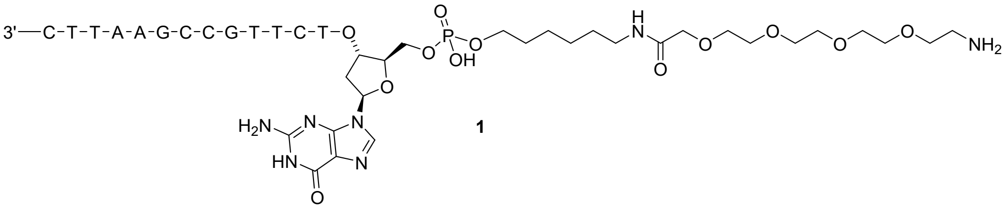

1

BPC:

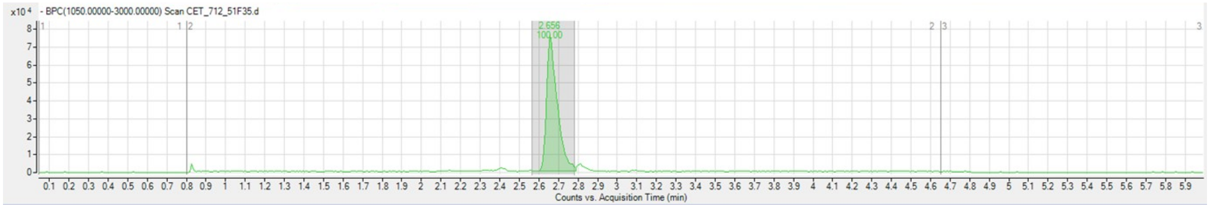

MS:

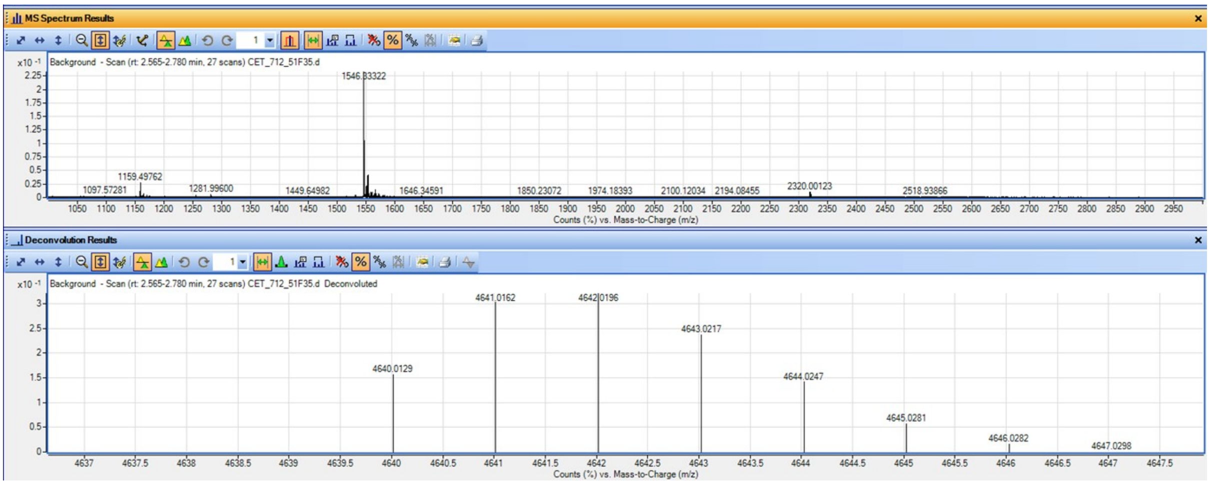

Validation experiment: Conversion >95%. **MS** (ESI-) expected 4989.1 Da, found 4989.1 Da

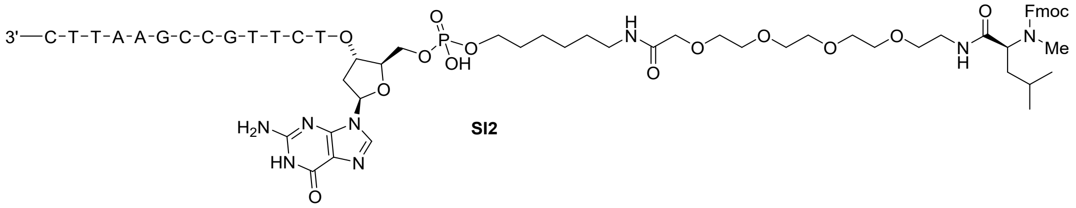

SI2

BPC:

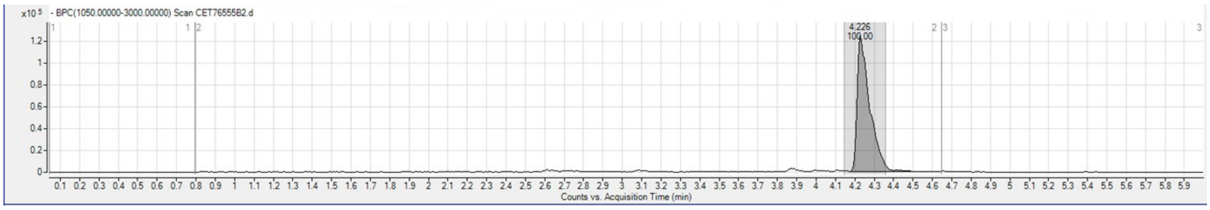

MS:

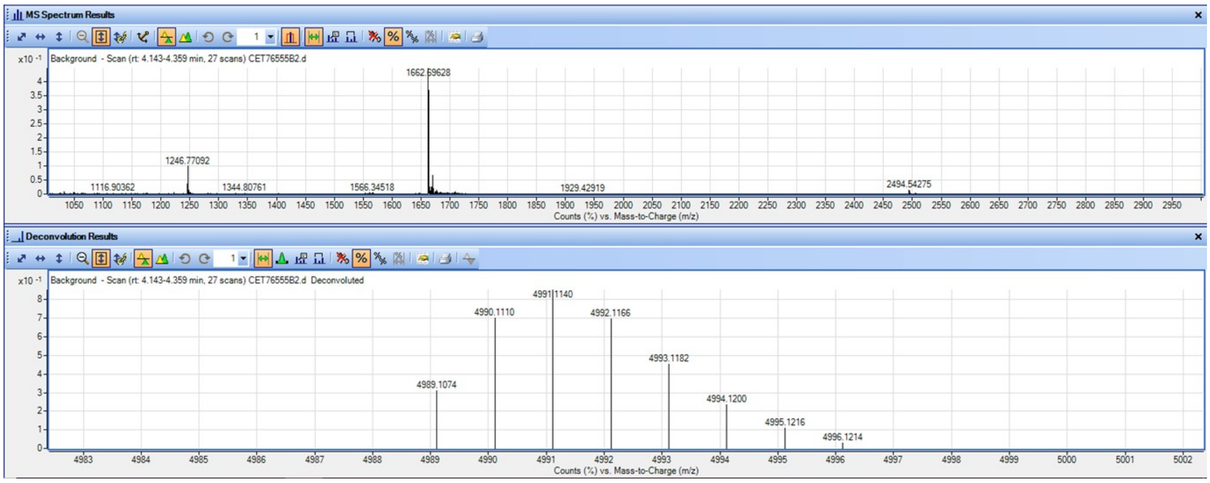

Validation experiment: Conversion >95%. **MS** (ESI-) expected 5018.1 Da, found 5018.1 Da

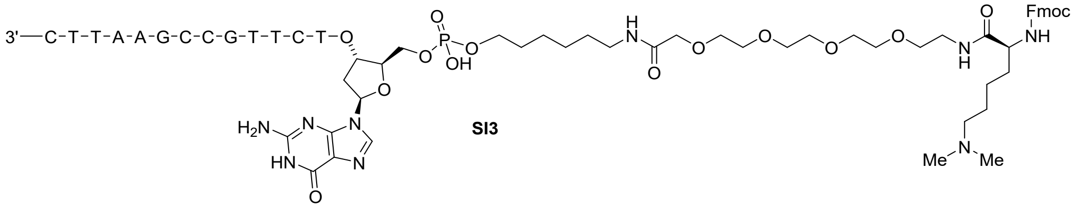

SI3

BPC:

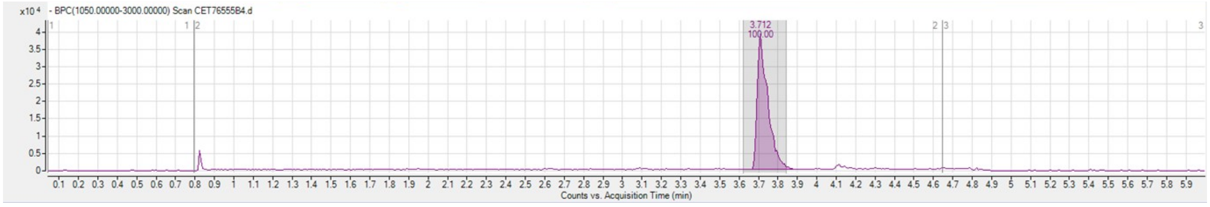

MS:

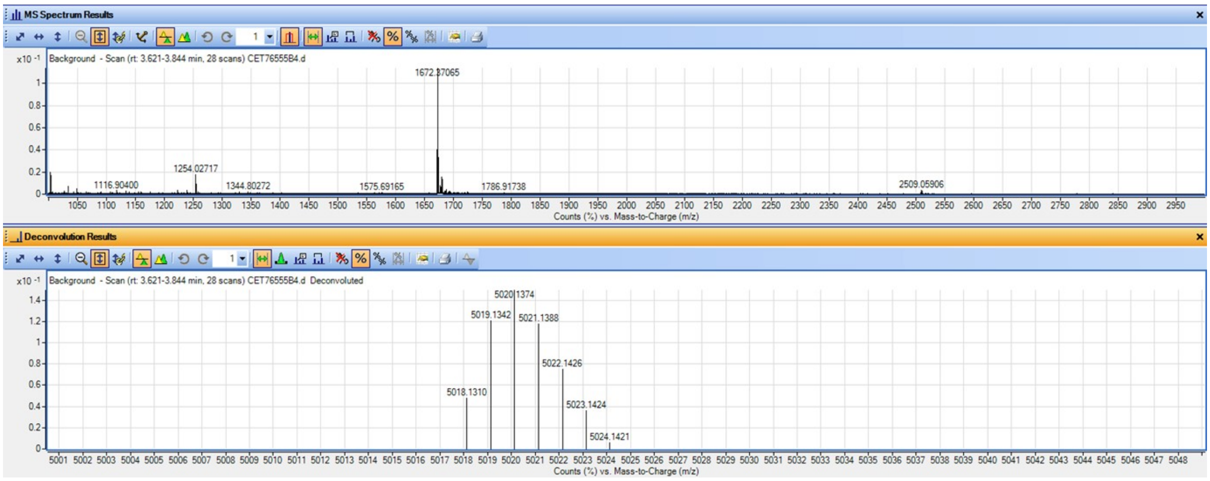

Validation experiment: Conversion >95%. **MS** (ESI-) expected 4959.1 Da, found 4959.1 Da

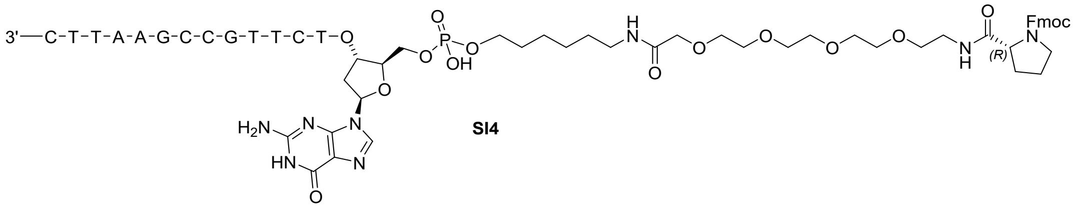

SI4

BPC:

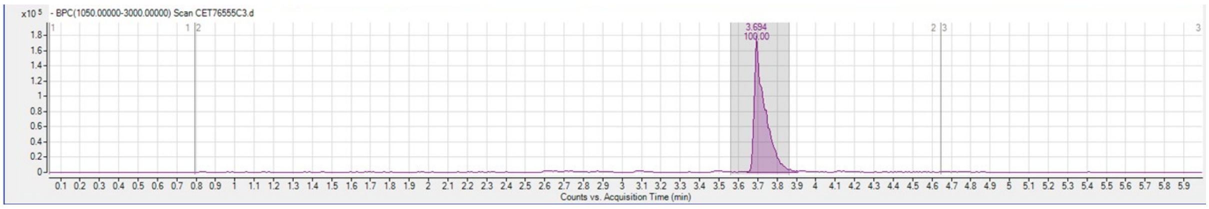

MS:

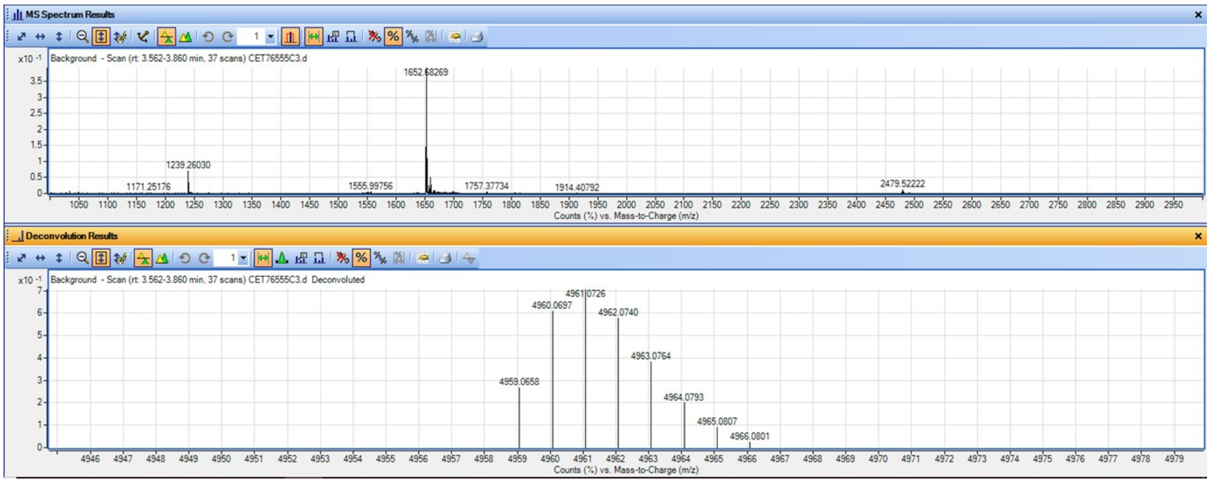

Validation experiment: Conversion >95%. **MS** (ESI-) expected 4933.0 Da, found 4933.1 Da

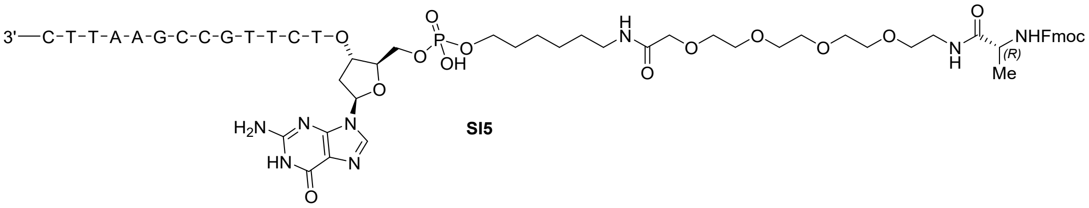

SI5

BPC:

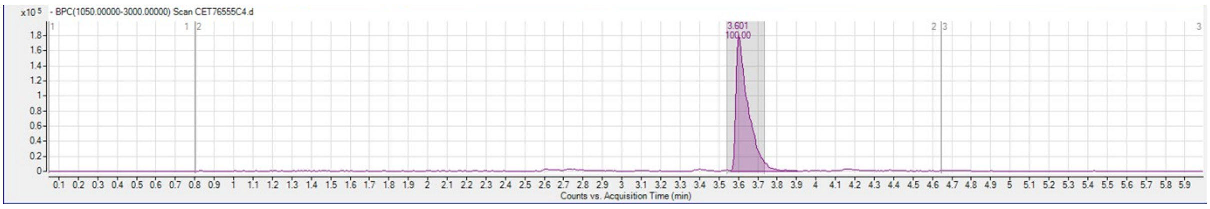

MS:

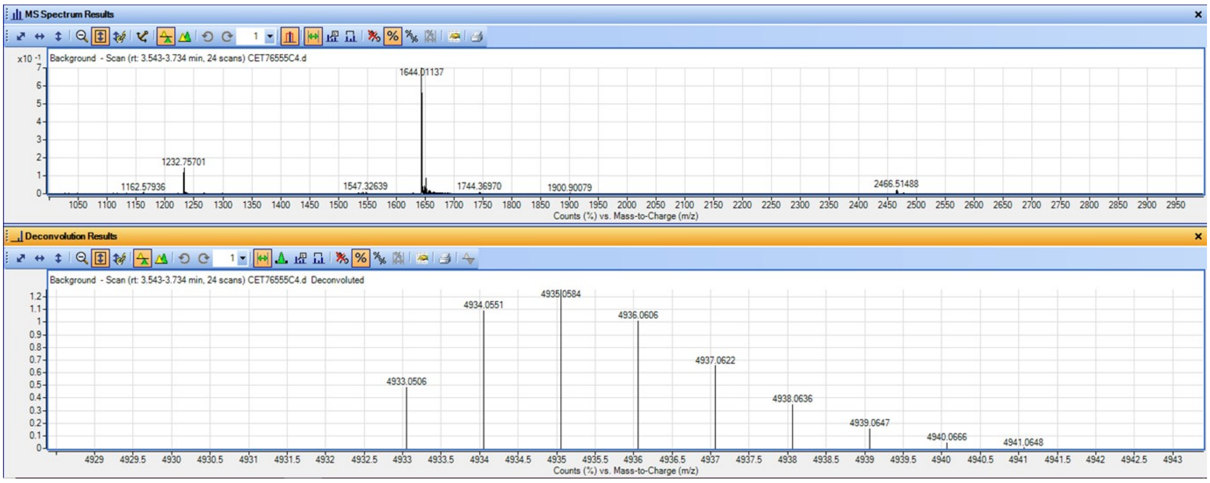

Validation experiment: Conversion 84%. **MS** (ESI-) expected 4961.1 Da, found 4961.1 Da

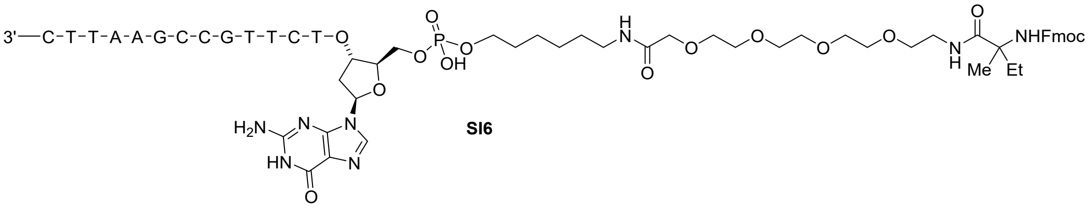

BPC:

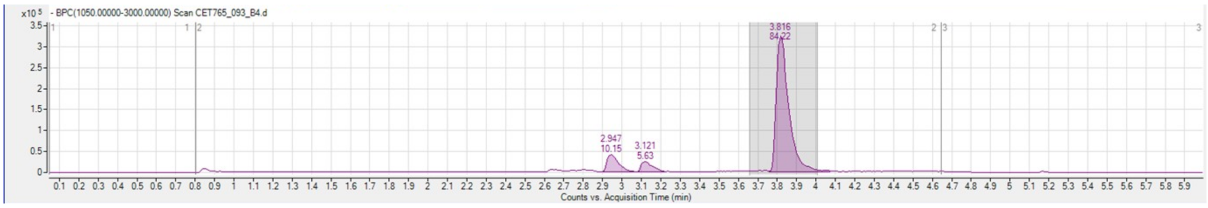

MS:

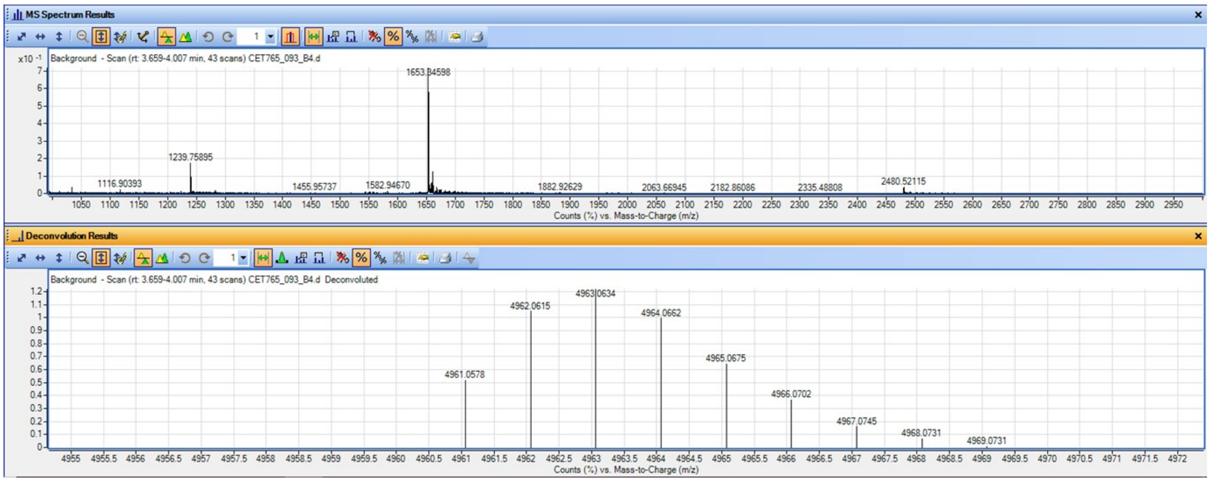

Validation experiment: Conversion >95%. **MS** (ESI-) expected 4783.0 Da, found 4783.0 Da

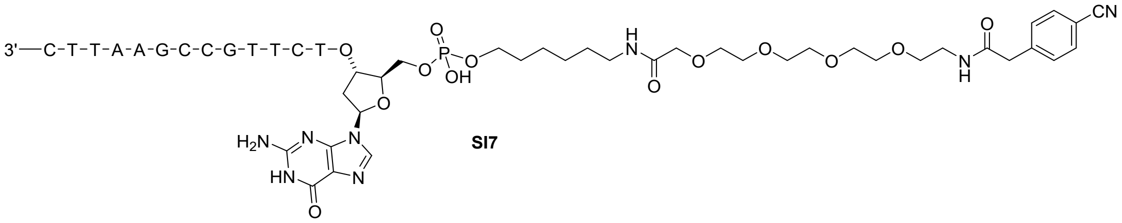

SI7

BPC:

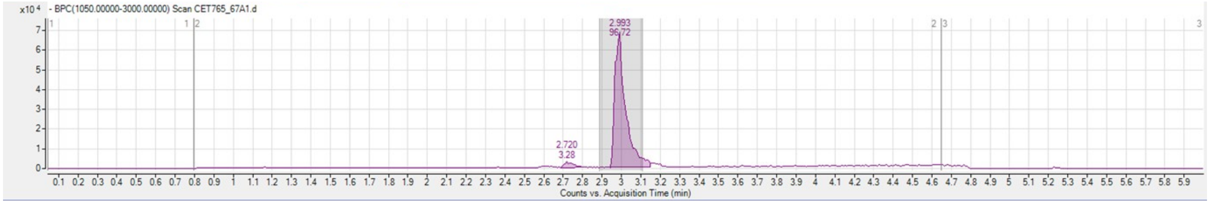

MS:

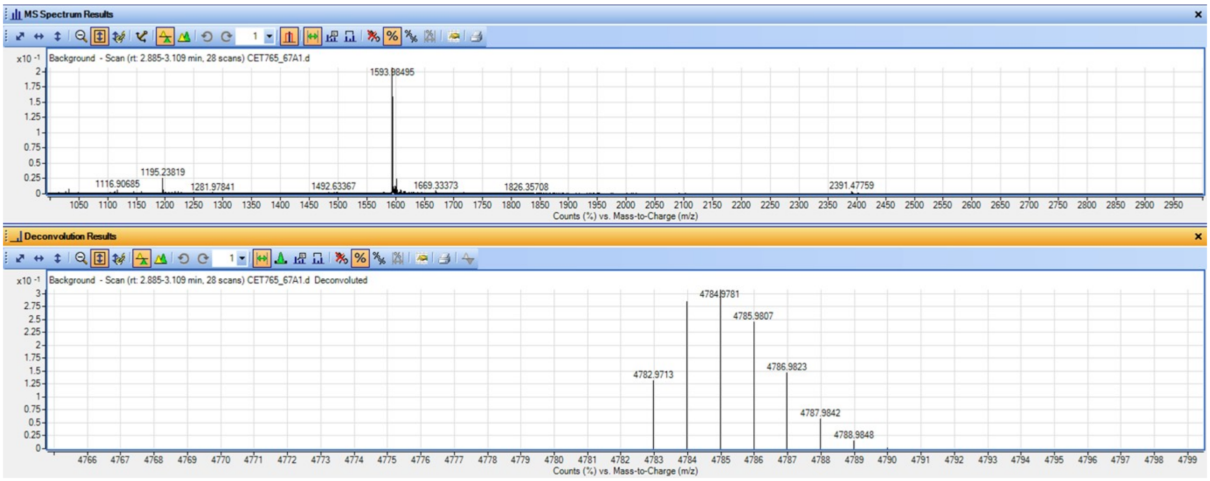

Validation experiment: Conversion >95%. **MS** (ESI-) expected 4811.0 Da, found 4811.0 Da

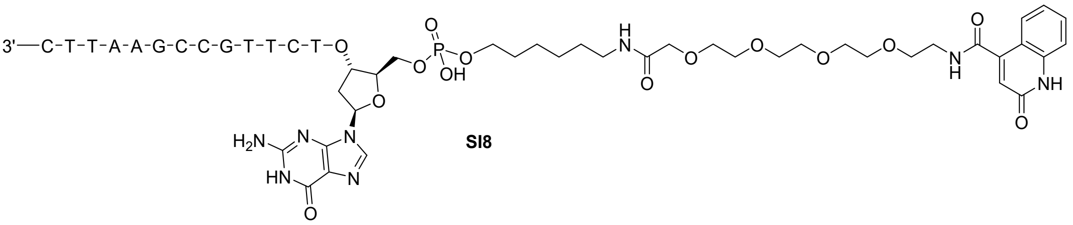

SI8

BPC:

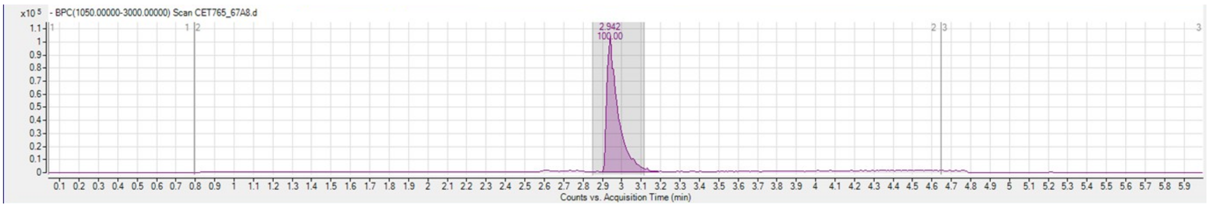

MS:

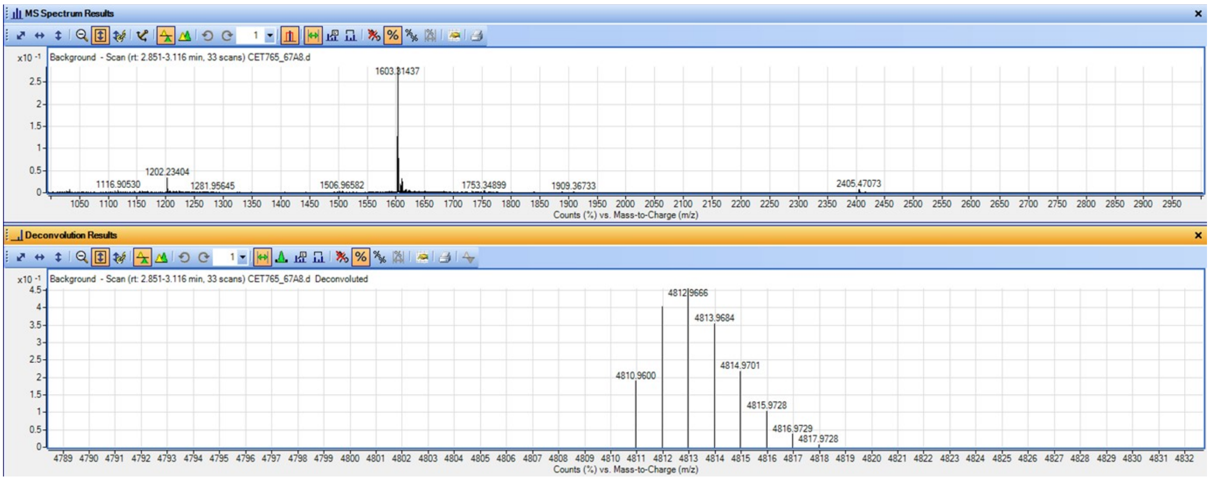

Validation experiment: Conversion >95%. **MS** (ESI-) expected 4795.0 Da, found 4795.0 Da

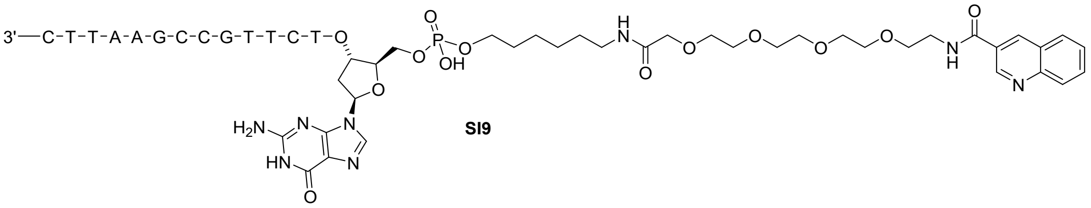

SI9

BPC:

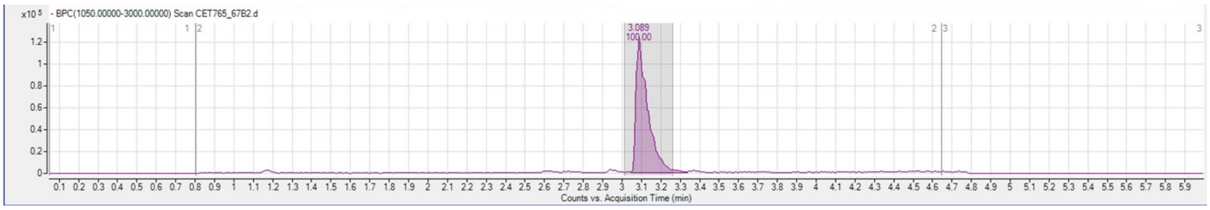

MS:

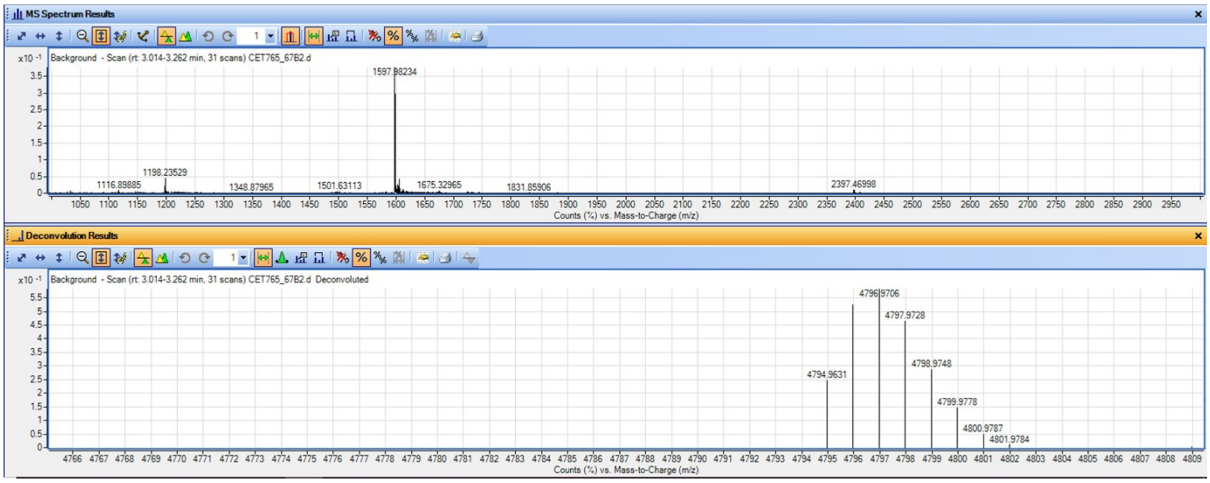

Validation experiment: Conversion 81%. **MS** (ESI-) expected 4960.0 Da, found 4960.0 Da

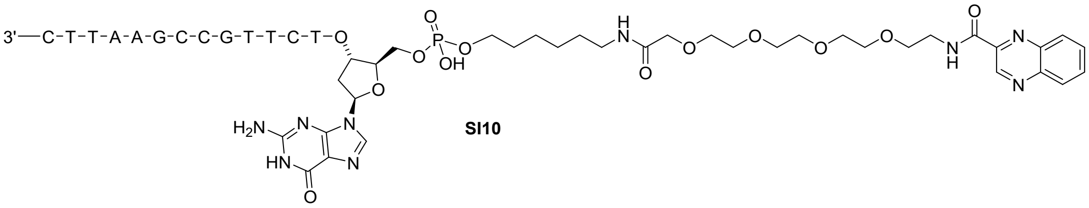

SI10

BPC:

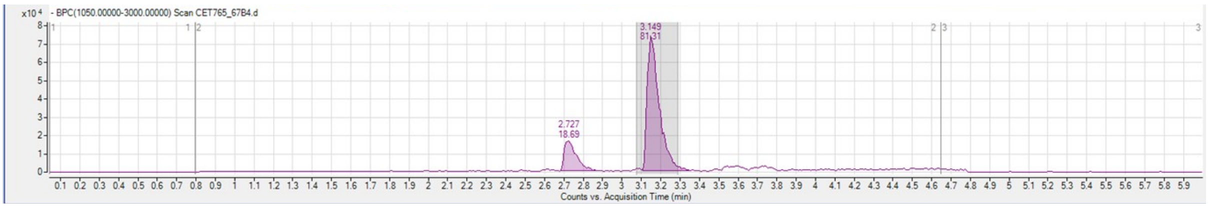

MS:

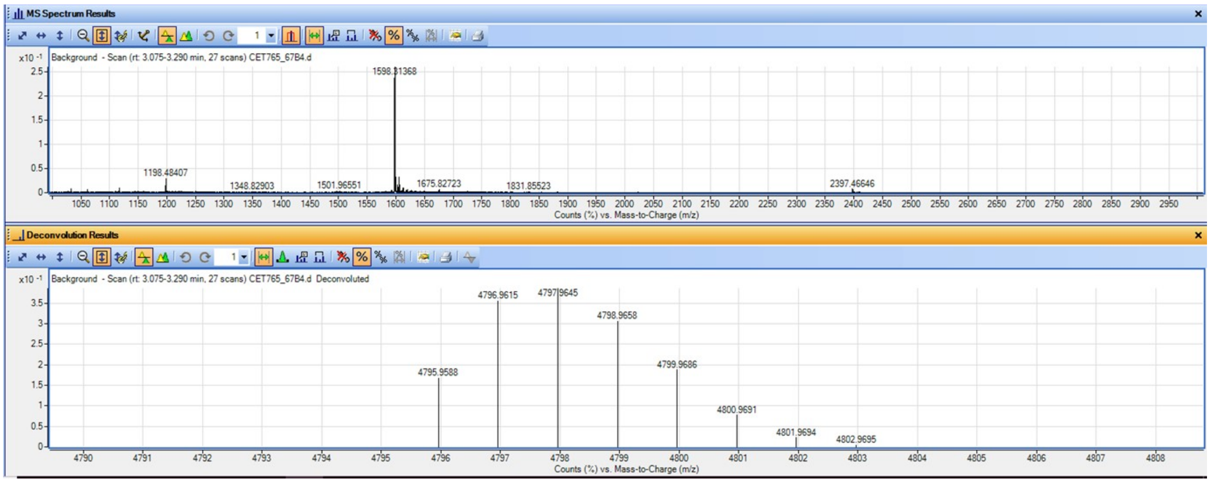

Validation experiment: Conversion >95%. **MS** (ESI-) expected 4775.0 Da, found 4775.0 Da

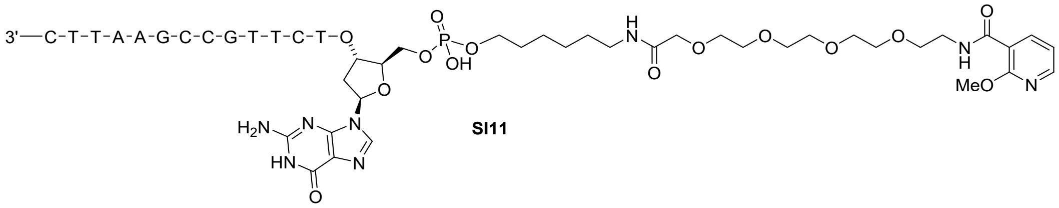

SI11

BPC:

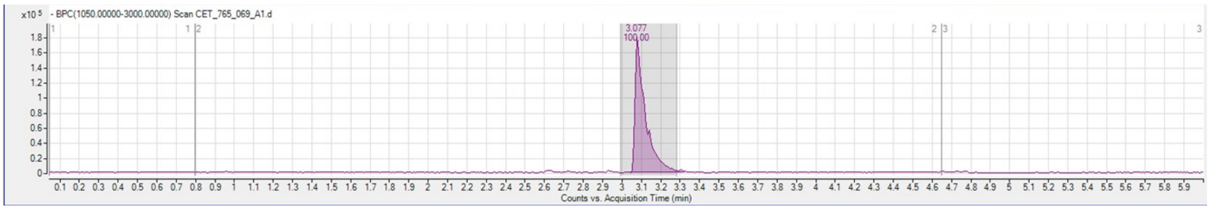

MS:

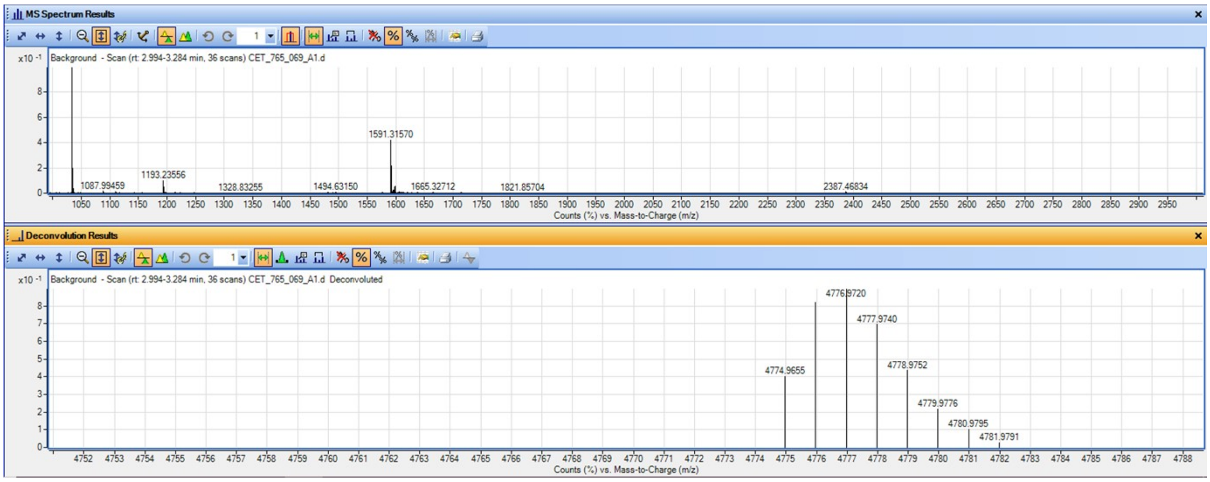

References

(1) Fan, L.; Yu, Y.; Jayne, C.; Frost, J. R.; Scott, J. D. Synthesis of DNA-Encoded Macrocyclic Peptides via Nitrile-Aminothiol Click Reaction. *Org Lett* 2023, **25** (44), 8038–8042.

(2) Hudson, L.; Mason, J. W.; Westphal, M. V.; Richter, M. J. R.; Thielman, J. R.; Hua, B. K.; Gerry, C. J.; Xia, G.; Osswald, H. L.; Knapp, J. M.; Tan, Z. Y.; Kokkonda, P.; Tresco, B. I. C.; Liu, S.; Reidenbach, A. G.; Lim, K. S.; Poirier, J.; Capece, J.; Bonazzi, S.; Gampe, C. M.; Smith, N. J.; Bradner, J. E.; Coley, C. W.; Clemons, P. A.; Melillo, B.; Hon, C. S. Y.; Ottl, J.; Dumelin, C. E.; Schaefer, J. V.; Faust, A. M. E.; Berst, F.; Schreiber, S. L.; Zécri, F. J.; Briner, K. Diversity-Oriented Synthesis Encoded by Deoxyoligonucleotides. *Nat Commun* 2023, **14** (1).
